# Structural and Functional Characterisation of a Metagenome‐Derived CYP108D18 Reveals an Unusual Dioxygen‐Bound State and Selective Hydrocarbon Hydroxylation

**DOI:** 10.1111/1751-7915.70451

**Published:** 2026-09-30

**Authors:** Simran Kundral, Peter D. Giang, Alicia M. Kirk, Julia B. Buczynski, Kasun S. A. Arachchige, Sunil K. Khare, Paul V. Bernhardt, Paul N. Evans, Luke W. Guddat, Stephen G. Bell, James J. De Voss

**Affiliations:** ^1^ The University of Queensland ‐ Indian Institute of Technology Delhi (UQIITD) Research Academy New Delhi India; ^2^ School of Chemistry and Molecular Biosciences The University of Queensland St Lucia Queensland Australia; ^3^ Enzyme and Microbial Biochemistry Laboratory, Department of Chemistry Indian Institute of Technology Delhi New Delhi India; ^4^ Scuola Normale Superiore Pisa Italy; ^5^ Centre for Microscopy and Microanalysis The University of Queensland St. Lucia Queensland Australia; ^6^ Department of Biological Sciences Indian Institute of Science Education and Research Kolkata Mohanpur West Bengal India; ^7^ Australian Centre for Ecogenomics, School of Chemistry and Molecular Biosciences The University of Queensland St Lucia Queensland Australia; ^8^ School of Physics, Chemistry and Earth Sciences Adelaide University Adelaide South Australia Australia

## Abstract

Cytochrome P450 enzymes form a large superfamily of monooxygenases capable of oxidising diverse substrates. Among them, members of the CYP108 family are recognised for their role in monoterpenoid oxidation. In this study, we report the identification and characterisation of a new CYP108 family member, CYP108D18, from a metagenome‐assembled genome derived from microbial communities in a geothermal hot spring located in northern Queensland, Australia. Taxonomic binning associated CYP108D18 with a *Novosphingobium* species, a genus well‐known for its bioremediation potential. CYP108D18 was heterologously expressed in *Escherichia coli* , yielding 640 nmol L^−1^ of culture, and purified to homogeneity. The enzyme exhibited moderate thermostability, with a ^15^T_50_ value of 47°C ± 1°C. Spectroscopic substrate binding assays revealed that while CYP108D18 bound monoterpenoids similarly to other CYP108 enzymes, it showed higher affinity and pronounced larger heme spin‐state shifts with aromatic hydrocarbons such as phenylcyclohexane and phenanthrene. Subsequent in silico docking and spectral‐binding studies also suggested that CYP108D18 may accommodate larger plant‐derived sesquiterpenes, indicating a potential role in their metabolism. Reconstitution of CYP108D18 with spinach ferredoxin and ferredoxin reductase resulted in the oxidation of phenylcyclohexane to *trans*‐4‐phenylcyclohexanol. Molecular dynamics simulations and ONIOM (QM/MM) calculations supported selective hydrogen abstraction at the cyclohexane C4 position, rationalising *trans*‐4‐phenylcyclohexanol as the predominant product. The high‐resolution crystal structure of substrate‐free CYP108D18 revealed a bound dioxygen ligand positioned above the heme iron, an unusual feature among P450 structures. Both substrate‐free and phenylcyclohexane‐bound structures revealed a persistent open conformation, suggesting the absence of a canonical open‐to‐closed transition upon ligand binding. Spectroelectrochemical analysis showed a heme redox potential of −451 mV versus NHE for the substrate‐free CYP108D18, shifting subtly to −409 mV upon phenylcyclohexane binding, which contributes to reduced activity with non‐native [2Fe‐2S] ferredoxins.

## Graphical abstract

CYP108D18, identified from a hot spring metagenome, catalyses selective phenylcyclohexane hydroxylation. This P450 has an exceptionally low substrate‐bound heme redox potential. Crystal structures revealed an unusual dioxygen‐bound substrate‐free form and a persistent open active site upon substrate binding.

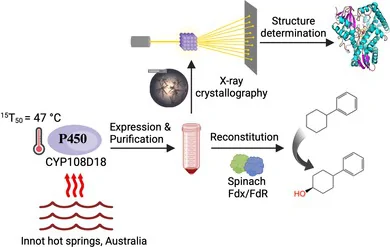

## 1 Introduction

Cytochrome P450 enzymes (P450s) are heme‐thiolate monooxygenases widely recognised as one of the most versatile biocatalysts in nature, able to catalyse a wide array of oxidative chemical transformations on a broad substrate range (Greule et al. 2018). In bacteria, P450s play important roles in both primary and secondary metabolism, contributing to the biosynthesis and biodegradation of compounds including terpenes, steroids, fatty acids and aromatic hydrocarbons. Their broad substrate scope and exceptional regio‐ and stereoselectivity have also made bacterial P450s attractive biocatalysts for industrial and pharmaceutical applications.

Terpenes are a large and structurally diverse class of natural products, classified by carbon number as monoterpenes (C_10_), sesquiterpenes (C_15_), diterpenes (C_20_) and higher terpenoids. Among these, the functionalisation and biodegradation of monoterpenes by P450s has been extensively studied (Ullah et al. 1990; Peterson et al. 1992; Hawkes et al. 2002; Bell and Wong 2007; Unterweger et al. 2016; Giang et al. 2022). Structural studies of the prototypical P450, P450_cam_ (CYP101A1) from *Pseudomonas putida* , both in the substrate‐free form (Poulos et al. 1986) and the D‐camphor‐bound form (Poulos et al. 1985, 1987), have greatly informed the current understanding of P450 structure and function. Additionally, the ferrous‐oxy intermediate of CYP101A1 has been crystallised and its structure solved (Schlichting et al. 2000; Nagano and Poulos 2005). Such ferrous‐oxy complexed structures have also been determined for two other P450s, P450eryF (Nagano et al. 2005) and CYP158A2 (Zhao et al. 2005).

The CYP108 family comprises functionally diverse bacterial class I P450 enzymes found in genera including *Pseudomonas*, *Novosphingobium* and *Rhodococcus*. Although initially associated with monoterpene oxidation through CYP108A1 (Peterson et al. 1992), characterised members of this family also bind aromatic and polycyclic aromatic hydrocarbons (Bell and Wong 2007). *Novosphingobium* species are particularly notable for their metabolic diversity, and numerous P450 genes in *N. aromaticivorans* have already been identified (Bell and Wong 2007). Many of these accept diverse substrates such as mono‐ and sesquiterpenoids, aromatic hydrocarbons, steroids and linear alkanes. One such enzyme, CYP108D1, a homologue of CYP108A1, exhibits tight binding to aromatic hydrocarbons containing up to three aromatic rings, such as phenylcyclohexane, phenanthrene, biphenyl and fluorene, while showing weaker affinity for monoterpenes (Bell et al. 2012). Although the conspecific ArR‐Arx class I electron transfer system effectively supports the activity of many *N. aromaticivorans* P450s (Bell et al. 2010; Yang et al. 2010), it displays lower activity with CYP108D1. Another CYP108 family member, CYP108J1 from *Rhodococcus* sp. P14, oxidises a broad range of polyaromatic hydrocarbons (PAHs), including phenanthrene, anthracene, biphenyl and benz[*a*]anthracene (Luo et al. 2016). It is co‐located with redox partner genes *fdx* (ferredoxin) and *hcaD* (ferredoxin reductase) within the same gene cluster, suggesting their involvement in supporting CYP108J1‐mediated PAH degradation. CYP108A1 from *Pseudomonas* sp. was the first in the CYP108 family to be crystallised in its substrate‐free form (Hasemann et al. 1994), and more recently, in its substrate‐bound (with α‐terpineol) form, and both structures were solved. Similarly, a substrate‐free crystal structure of CYP108D1 from *N. aromaticivorans* has also been determined (Bell et al. 2012).

Metagenomics has emerged as a powerful approach for discovering novel and robust enzymes by mining DNA sequences directly derived from microbial communities (Scherlach and Hertweck 2021). However, the identification and subsequent characterisation of P450 enzymes from Metagenome Assembled Genomes (MAGs) remain relatively scarce (Hogg et al. 2024). Recently, our group identified and characterised a thermophilic, self‐sufficient P450 monooxygenase (CYP116B305) from a metagenomic database generated using microbial biomass collected from the Innot hot springs in North Queensland, Australia (Kundral et al. 2025). These springs exhibit surface temperatures ranging from 62°C to 71°C and a neutral to alkaline pH (7.2–8.1). In this study, we report the identification and characterisation of another P450 enzyme, CYP108D18, associated with a *Novosphingobium* sp. 010 MAG, from the same metagenomic database of the Innot hot springs. Sharing 68% sequence identity with CYP108D1, CYP108D18 was hypothesised to be involved in the degradation of aromatic hydrocarbons and/or monoterpenes. In the absence of native redox partners, various surrogate systems were tested for their ability to support its activity. To gain structural insights into substrate binding, we solved the crystal structures of CYP108D18 in its substrate‐free form and in complex with phenylcyclohexane, at resolutions of 2.06 Å and 2.25 Å, respectively. The substrate specificity of the enzyme was also explored via in silico docking and binding experiments, the latter indicating a preference for sesquiterpenes and suggesting a biological role in the metabolism of these compounds.

## 2 Materials and Methods

### 2.1 General

All the chemicals and reagents used in this study were of analytical grade and obtained from Sigma‐Aldrich unless stated otherwise. Bacto‐tryptone was purchased from Thermo Fisher Scientific (Gibco, AU).

### 2.2 Identification and Phylogenetic Analysis of CYP108D18

Sample collection and metagenomic sequencing of the Innot Hot Springs were performed as previously described (Kundral et al. 2025). A 1308 bp open reading frame encoding CYP108D18 was identified within a MAG designated *Novosphingobium* sp. SE0864_S24.metabat2_refined.010 assembled from the metagenomic dataset. Sequence homologies were determined using the BLAST tool in UniProt (The UniProt Consortium et al. 2025). Amino acid sequences were aligned using Clustal Omega (Sievers et al. 2011), and a phylogenetic tree was constructed using the UPGMA method in MEGA11 software (Tamura et al. 2021). Evolutionary distances, expressed as the number of amino acid substitutions per site, were calculated using the Poisson correction method.

### 2.3 Heterologous Expression and Purification of CYP108D18

The *CYP108D18* gene sequence was codon‐optimised for *E. coli* , commercially synthesised and cloned into pET‐28a(+) plasmid by Twist Biosciences, USA, to form a pET‐28a(+)/*CYP108D18* construct with an incorporated N‐terminal 6× His tag. This plasmid was then transformed into *E. coli* BL21(DE3) competent cells and plated on Luria‐Bertani (LB) agar containing kanamycin (50 μg mL^−1^). The following day, single‐colonies were inoculated into LB medium (5 mL) containing kanamycin (50 μg mL^−1^) and incubated at 37°C overnight. The overnight seed culture was then used to inoculate Terrific Broth (TB, 500 mL) medium containing kanamycin (50 μg mL^−1^) and cells were grown at 37°C until an O.D. ~0.8 was observed. Cultures were then cooled down to 25°C, followed by addition of isopropyl β‐D‐1‐thiogalactopyranoside (IPTG, 1 mM) to induce protein expression. δ‐Aminolevulinic acid (δ‐ALA, 0.5 mM) was added concurrently to promote heme biosynthesis. The cultures were incubated for a further 16 h at 25°C. The cells were harvested by centrifugation at 5000 × *g* for 20 min, 4°C.

All of the following steps for protein purification were carried out at 4°C, unless otherwise stated. The cell‐pellet was resuspended in Tris‐HCl buffer (100 mM, 50 mM NaCl, 5% glycerol, pH 7.4) supplemented with phenyl methyl sulfonyl fluoride (PMSF, 200 μM), DL‐dithiothreitol (DTT, 0.5 mM) and lysozyme (0.1 g L^−1^). Cells were lysed by sonication (Branson Sonifier 450) with 3 cycles of 30 s each (1 s on and 1 s off, 30% amplitude), followed by centrifugation (20,000 × *g*, 50 min) to remove the cell debris. The collected cell lysate was diluted 1/3 with Tris‐HCl buffer (100 mM, pH 7.4) and loaded onto a Ni‐NTA column (5 mL, HisTrap FF, Cytiva) pre‐equilibrated with the same buffer and connected to an FPLC ÄKTA pure system. Elution was performed using a stepwise gradient of 20–150 mM L‐histidine, and fractions were collected automatically and analysed for purity using UV‐visible spectroscopy. Fractions with a Reinheitszahl (RZ, absorption ratio A_417nm_/A_280nm_) > 1 were combined, concentrated using ultrafiltration (Amicon filtration units), and resuspended in Tris‐HCl buffer (50 mM, 5% glycerol, pH 7.4) for storage at −80°C until further use. The concentration of purified CYP108D18 was measured using an extinction coefficient of ε_450_ = 91 mM^−1^ cm^−1^, following the protocol for the CO‐difference assay (Guengerich et al. 2009).

### 2.4 Determination of Thermostability

The thermostability of CYP108D18 was determined by measuring the residual intact CYP108D18 by CO‐difference assay after incubating the purified protein at elevated temperatures compared to the protein retained at 4°C. A solution containing purified CYP108D18 (1 μM) in Tris–HCl buffer (50 mM, pH 7.4) was incubated at various temperatures (30°C–70°C) for either 15 or 60 min. The residual CO‐binding activity was then measured and plotted against the temperature. The obtained data were fitted to a Hill‐type equation (Equation 1) using GraphPad Prism to extract T_50_ values (T_m_) for both the time points. *T*_50_ refers to the temperature at which 50% of CO‐binding remains after the incubation, *y* refers to the residual CO‐binding activity at a given temperature *T*, *y*_*0*_ refers to the maximum CO‐binding activity of non‐incubated protein solution, and *h* is the Hill coefficient.

(1) $$y=y_{0}-\frac{\left(y_{0}\cdot T^{h}\right)}{\left(T_{50}^{h}+T^{h}\right)}$$

### 2.5 Substrate Binding Analysis

To determine the dissociation constant (K_D_ ) for substrates with CYP108D18, difference spectroscopy was performed between a solution of CYP108D18 (1 μM) and a solution of the same enzyme with an increasing concentration of the compound of interest (0–100 μM). The absolute absorbance difference between 418 nm and 393 nm was plotted against the increasing substrate concentration and K_D_ was calculated using the typical hyperbolic equation (Equation 2). However, for tight binding substrates, a quadratic equation was applied (Equation 3) (Morrison 1969), where *A* is the absorbance difference between 393 nm and 418 nm, *A*_max_ is the maximal absorbance difference between 393 and 418 nm, [ *E*_*T*_ ] is the concentration of CYP108D18, and [ *S*_*T*_ ] is the total substrate concentration

(2) $$\begin{array}{c} V=\frac{∆V\max\times \left[S\right]}{K_{D}+\left[S\right]} \end{array}$$

(3) $$\begin{array}{c} \mathrm{\Delta A}=\Delta A_{\max}\frac{\left(\left[E_{T}\right]+\left[S_{T}\right]+K_{D}\right)-\sqrt{{\left(\left[E_{T}\right]+\left[S_{T}\right]+K_{D}\right)}^{2}-4\left[E_{T}\right]\left[S_{T}\right]}}{2\left[E_{T}\right]} \end{array}$$

The heme iron spin state shift was estimated by comparison with a set of spectra generated from the sum of the appropriate percentages of the spectra of the substrate‐free form (> 95% low spin, Soret peak at 418 nm) and phenylcyclohexane bound form (95% high spin, Soret peak at 390 nm) of CYP108D1.

### 2.6 Redox Characterisation

A spectroelectrochemical quartz cell (Pine instruments, 1.7 mm path length) containing a gold ‘honeycomb’ working electrode and a counter electrode with a separate Ag/AgCl reference electrode (calibrated with quinhydrone E′ + 288 vs. normal hydrogen electrode (NHE) at pH 7) was utilised. The reaction cell contained approximately 600 μL of solution (50 mM Tris–HCl, pH 7.4), with substrate‐free (30 μM CYP108D18) or substrate‐bound (30 μM CYP108D18 and 100 μM of phenylcyclohexane) P450. The following mediator complexes (36 μM) were used: [Fe(*trans*‐diammac)]^3+^, [Co(AMMEN_4_S_2_sar)]^3+^, [Co(AMMEN_5_Ssar)]^3+^, [Co(sep)]^3+^, [Co(AMMEsar)]^3+^, [Co(ClMeClabsar)]^3+^, [Co(cis‐diammac)]^3+^ and [Co(*trans*‐diammac)]^3+^ (Scheme S1). These mediator complexes provide redox buffering across −35 to −555 mV versus NHE and have small extinction coefficients (ε < 300 M^−1^ cm^−1^), which avoids any significant contribution to the UV‐visible absorption spectra observed (Bernhardt et al. 2006).

Potential dependent UV‐visible absorption spectra were measured over 300–800 nm (Agilent 8453 diode array UV‐visible spectrophotometer) with temperature controlled by a Huber Ministat 230 Cooling Bath Circulation Thermostat. Potentials were set and controlled by a potentiostat (Gamry Interface 1010) in constant potential coulometry mode.

Absorption spectra were recorded when absorbance changes ceased (2–10 min). Spectra were measured at 25 mV intervals with decreasing potential followed by interspaced 25 mV intervals with increasing potential, resulting in 12.5 mV intervals with reversibility. All wavelengths of the potential‐dependent absorbance spectra were analysed and modelled with Reactlab Redox (Maeder and King 2016) using a single (Equation 4) electron transfer model. In this model, *A*_*ox*_ and *A*_*red*_ represent the limiting absorbances of the protein in its fully oxidised and reduced states, respectively, at a single wavelength and *Abs* denotes the absorbance at that same wavelength under an applied potential *E*.

(4) $$\begin{array}{c} \mathrm{Abs}=\frac{\left(A_{\mathrm{ox}}10^{\frac{n\left(E-E^{0}\right)}{59}}+A_{\mathrm{red}}\right)}{1+10^{\frac{n\left(E-E^{0}\right)}{59}}} \end{array}$$

### 2.7 NAD(P)H Consumption Assays

The NAD(P)H oxidation rates were determined using reactions prepared in Tris–HCl buffer (50 mM, pH 7.4) containing purified CYP108D18 (0.5 μM), ferredoxin (4 μM or 5 μM) and ferredoxin reductase (1 μM), resulting in a 1:8:2 or 1:10:2 ratio (P450:Fdx:FdR). Substrates dissolved in 100% ethanol were added to the reactions (final concentration 1 mM). Catalase (1 μM) was also added to degrade any hydrogen peroxide formed from uncoupling processes. NAD(P)H (200 μM) was added last to initiate the reaction. The rate of NAD(P)H consumption was measured by monitoring the disappearance of absorbance at 340 nm over 1 min, using an extinction coefficient of NAD(P)H, ε_340nm_ = 6220 M^−1^ cm^−1^.

### 2.8 In Vitro Turnover Experiments

In vitro catalytic turnover experiments were performed in Tris–HCl buffer (50 mM, pH 7.4) containing purified CYP108D18 (0.5 μM), ferredoxin (4 μM or 5 μM), ferredoxin reductase (1 μM), catalase (1 μM) and substrate (1 mM). Reactions were started by the addition of NAD(P)H (1 mM) and stirred at room temperature (~25°C) for 2 h, followed by extraction with ethyl acetate (3 × 1 mL). The organic phase was collected after each extraction, combined and analysed by gas chromatography–mass spectrometry (GC–MS; Zebron ZB‐5MS column 30 m × 0.25 mm × 0.25 μm). The GC temperature program involved an initial hold at 50°C for 5 min, followed by a ramp of 16°C per min to 250°C, which was then maintained for 8 min. Products were identified by co‐elution with synthetic standards or by comparison to a GC–MS spectrometric library (Wiley, NIST).

### 2.9 Crystallisation and Structure Determination

Purified CYP108D18 protein was concentrated to 28 mg mL^−1^ in Tris–HCl buffer (20 mM, pH 7.4). Crystallisation was performed using the hanging‐drop vapour diffusion method at 20°C. Drops were formed by mixing 150 nL of protein solution (28 mg mL^−1^) and 150 nL of reservoir solution containing 0.04 M sodium citrate (pH 2.3), 0.06 M 1,3‐bis[tris(hydroxymethyl)methylamino]propane (BTP, pH 9.7) and 20% (w/v) PEG 3350. The drops were then equilibrated against 200 μL of the same reservoir solution. Crystals of the CYP108D18 in complex with phenylcyclohexane were obtained using 0.2 M sodium malonate and 20% (w/v) PEG 3350 as the reservoir solution. Diffraction‐quality crystals appeared within 1–2 weeks.

X‐ray diffraction experiments were conducted at the MX2 beamline of the Australian Synchrotron (McPhillips et al. 2002). The collected datasets were processed through indexing, integration, scaling and merging using XDS (Kabsch 2010). For structure determination, the phases were solved by the molecular‐replacement (MR) method using the program Phaser in the CCP4 suite (McCoy et al. 2007). The structure of CYP108D1 (PDB: 3TKT) was used as the search model. Model construction and manual adjustments were performed in Coot (v0.9.8.95) (Emsley and Cowtan 2004), followed by iterative refinement using PHENIX (Adams et al. 2010), respectively. Key crystallographic and refinement parameters are summarised in Table 1. The final atomic coordinates and structure factors of both the substrate‐free and phenylcyclohexane‐bound structures have been deposited in the Protein Data Bank (PDB) and will be released upon publication under accession codes 27IS and 27DK, respectively.

**TABLE 1:** X‐ray crystallography data collection and refinement statistics.

	Substrate‐free	Phenylcyclohexane‐bound
Data‐collection statistics		
Space group	*C* 2_1_	*C* 2_1_
Unit cell lengths (Å)	*a* = 63.97, *b* = 92.11, *c* = 78.71	*a* = 63.57, *b* = 92.57, *c* = 78.85
Unit cell angles (°)	*α* = *γ =* 90, *β =* 96.66	*α* = *γ =* 90, *β =* 97.02
Wavelength (Å)	2.06	2.25
Resolution (Å)	46.05–2.06 (2.12–2.06)^a^	46.28–2.25 (2.32–2.25)
⟨I/*σ*(I)⟩	11.8 (1.2)	5.6 (0.9)
Completeness (%)	99.4 (93.4)	98.7 (86.6)
Average multiplicity	6.8 (6.4)	6.6 (5.9)
*R*_pim_ (%)^b^	0.038 (0.480)	0.091 (0.661)
Structure‐refinement statistics		
Resolution (Å)	46.05 − 2.06	45.56 − 2.26
Average B factor (Å^2^)	50.02	52.91
R_work_/R_free_ (%)	22.45/27.10	24.01/28.77
R.m.s. deviation from ideal bond lengths (Å)	0.003	0.003
R.m.s. deviation from ideal bond angles (°)	0.625	0.580
Ramachandran favoured (%)	97.15	94.99
Ramachandran outliers (%)	0.00	0.48
Rotamer outliers (%)	0.29	0.29
Clash scores^c^	4.79	6.58

^a^ Values in parentheses are for the outer resolution shell.

^b^ $R_{p.i.m.}=\sum_{\mathrm{hkl}}\;{\left[\frac{1}{\left[N\left(\mathrm{hkl}\right)-1\right]}\right]}^{1/2}\sum_{i}\left|I_{i}\left(\mathrm{hkl}\right)-\left(I\left(\mathrm{hkl}\right)\right)\right|/\sum_{\mathrm{hkl}}\sum_{i}I_{i}\left(\mathrm{hkl}\right)$ where *I*_*i*_(*hkl*) is the observed intensity and *I*(*hkl*) is the average intensity obtained from multiple observations of symmetry related reflections.

^c^ Clashscore is defined as the number of bad overlaps ≥ 0.4 Å per thousand atoms.

### 2.10 Molecular Docking

Molecular docking was performed using AutoDock Vina version 1.1.2 (Trott and Olson 2010). The crystal structure of CYP108D18 was prepared by removing solvent molecules and the bound ligand while retaining the heme cofactor. Polar hydrogen atoms and partial charges were added to the protein. Ligand structures were energy‐minimised and converted to PDBQT format with Gasteiger charges and rotatable bonds assigned. A docking grid covering the active‐site cavity above the heme was used. Following docking, the predicted poses were visually inspected using PyMOL. Poses were selected based on their orientation relative to the heme iron, the proximity of a potentially oxidisable carbon atom and the absence of major steric clashes. The docking procedure was evaluated by redocking phenylcyclohexane and comparing its predicted pose with the crystallographically observed binding mode. Docking scores are reported in kcal/mol.

### 2.11 Molecular Dynamics (MD) Simulations

MD simulations of phenylcyclohexane (PCH) bound CYP108D18 in the active oxidant form (Compound I) were performed with Amber 20 (Case et al. 2023) using pmemd (Salomon‐Ferrer et al. 2013). In order to model the unresolved *F*‐*G* loop, the starting structure was generated using Alphafold3 (webserver) (Abramson et al. 2024) including placement of the heme. The protonation states of the residues were assigned using the H++ 3.0 webserver (Anandakrishnan et al. 2012) followed by manual inspection. PCH was manually docked through alignment with the crystal structure (PDB 27DK). Non‐clashing crystallographic waters were also retained. There was good alignment of the protein backbone across the majority of the AlphaFold and crystal structures (RMSD 0.5 Å residues 1–178, 241–436); however, deviations in the *F* and *G* helices were noted (RMSD 1.8 Å). These deviations were minimised during equilibration using a series of distance and torsional restraints to achieve closer structural agreement with the crystal structure (adjusted *F*, *G* helices RMSD 1.1 Å, Figure S1). Parameters for the thiolate coordinated heme (Cpd I) were taken from Cheatham and coworkers (Shahrokh et al. 2012). Parameters for PCH were generated with AmberTools25 using *antechamber* with AM1‐BCC charges and the General Amber Force Field (gaff2) (Wang et al. 2006). The Amber 14 force field with Stony Brook modifications (ff14SB) (Maier et al. 2015) was employed for the protein. Simulations were performed as per previously reported methodology (Coleman et al. 2022). See Appendix S1 for complete details.

### 2.12 ONIOM Calculations

ONIOM (Vreven et al. 2006) (so‐called Our own *N*‐layered Integrated molecular Orbital and molecular Mechanics) calculations were performed using Gaussian 16 (rev. c01) (Frisch et al. 2016). A representative structure was obtained from the major cluster identified in MD simulations. Ions and water molecules beyond the first solvation shell were stripped. The high layer comprised the phenylcyclohexane substrate, the heme with propionate groups truncated and coordinating cysteine truncated to SMe (S‐methyl). Residues within 12 Å of the high layer were free to move while the remainder were frozen. Geometries, employing quad macro, were optimised for the quartet spin state at the ONIOM(B3LYP/BS1:AMBER(ff14SB)) level of theory with mechanical embedding, where BS1 applies the SDD basis set and pseudopotential to Fe and 6‐31G(d) to all other atoms. Frequency calculations were used to obtain zero‐point‐energy corrections and confirm the nature of the stationary point. Single‐point energy corrections were calculated at the ONIOM(B3LYP/Def2TZVP:AMBER (ff14SB)) level of theory. Transition state (TS) structures were initially located by scanning H‐O distances starting from the reactant complex before full TS geometry optimisation. Molecular mechanics (MM) energy decomposition was performed alchemically through a series of single‐point ONIOM (MM:MM) calculations setting the parameters for each residue to zero in turn using dummy atom definitions. Images were generated using UCSF ChimeraX (Meng et al. 2023).

## 3 Results and Discussion

### 3.1 Identification of CYP108D18 From Novosphingobium sp.

From the metagenomic data obtained from Innot hot springs, in North Queensland, Australia, a putative cytochrome P450 encoding gene (*CYP108D18*), 1308 bp in length, was identified within a genome classified under the *Novosphingobium* genus. An alignment revealed that this gene shared over 40% protein sequence identity with members of the CYP108 family, including CYP108A1 and CYP108D1 (Figure 1, Figure S2). CYP108D18 showed the highest protein sequence identity (68%) to CYP108D1 from *N. aromaticivorans* . Based on this similarity, the gene was classified as a new member of the subfamily and designated as CYP108D18 (Nelson 2009). Such a high sequence identity suggests that these enzymes may share similar substrate preferences, particularly towards aromatic hydrocarbons, as observed with CYP108D1.

**FIGURE 1:**
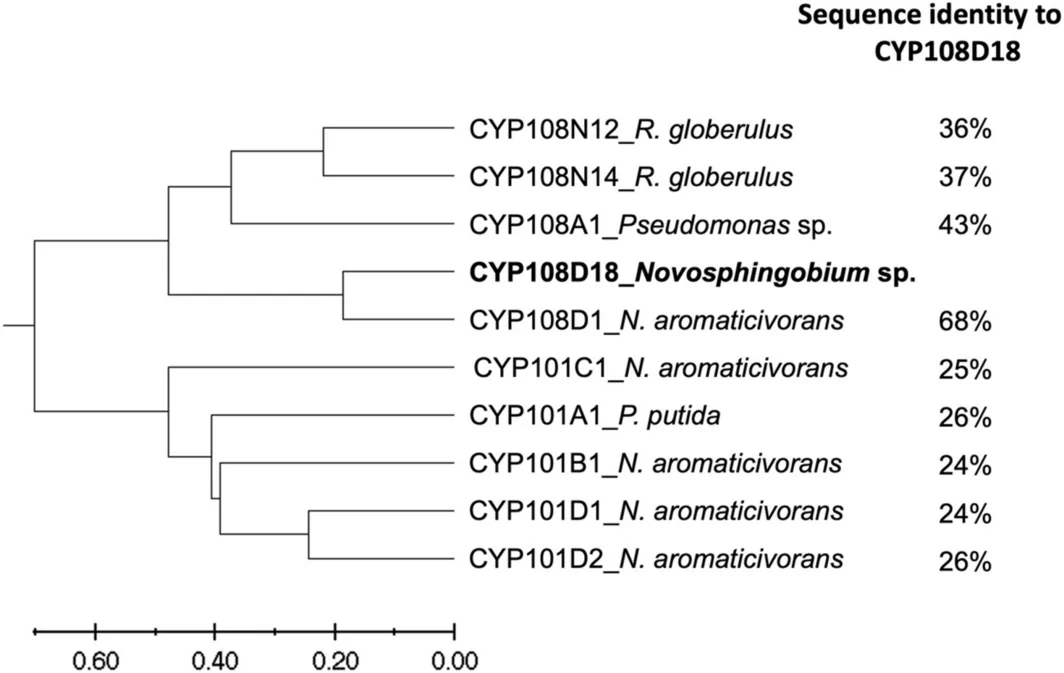
Phylogenetic analysis of the amino‐acid sequence of CYP108D18 with selected P450s from CYP108 and CYP101 families. Sequence alignment was performed using Clustal Omega, with evolutionary history inferred using the UPGMA method and visualised in MEGA11.

### 3.2 Heterologous Expression and Purification of CYP108D18

Various expression conditions were tested to optimise the heterologous production of CYP108D18 in *E. coli* . These included varying IPTG concentrations (0–1 mM), δ‐ALA concentrations (0.1–0.5 mM) and post‐induction temperatures (18°C–30°C). CO‐difference assays revealed that the highest expression level of CYP108D18, approximately 640 nmol L^−1^ of culture (corresponding to 32 mg L^−1^), was achieved in the presence of IPTG (1 mM) and δ‐ALA (0.5 mM) at 25°C. The overexpressed CYP108D18 was purified via affinity chromatography (~410 nmol L^−1^ of culture). The purity of the protein was initially assessed using UV‐visible spectroscopy by calculating the Reinheitszahl (RZ), defined as the ratio of absorbance at 417 nm to that at 280 nm (A_417_/A_280_), which yielded a value of 1.4 (Figure S3). Further confirmation of protein purity was obtained through SDS‐PAGE analysis, which showed a single band at ~50 kDa, consistent with the expected molecular weight of CYP108D18 (48 kDa) plus the N‐terminal His_6_‐tag and thrombin cleavage site (Figure S4).

Spectroscopic analysis of the purified CYP108D18 protein revealed characteristic features of an oxidised ferric cytochrome P450, including a Soret peak at 417 nm, and α and β bands at 569 nm and 535 nm, respectively (Figure 2a). Upon reduction with sodium dithionite, the Soret peak displayed an expected shift to 413 nm for the ferrous heme. Subsequent exposure to carbon monoxide resulted in a distinct absorbance maximum at 450 nm, confirming the formation of the CO‐bound, ferrous P450 complex and also that the purified enzyme was properly folded. A minor shoulder near 420 nm (P420 species) was also observed, suggesting the presence of a small fraction of denatured or conformationally altered protein.

**FIGURE 2:**
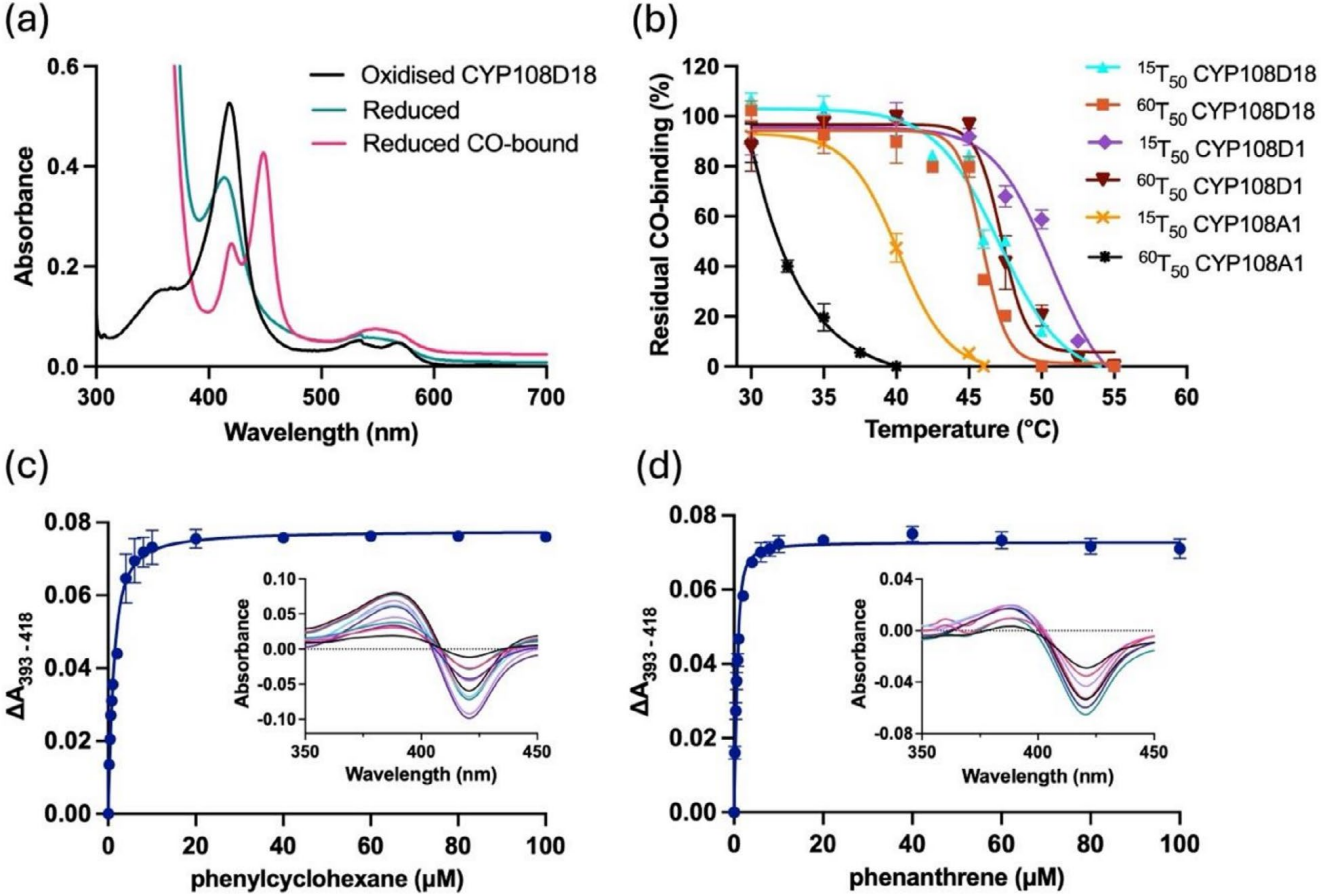
UV‐visible spectra (300–700 nm) of purified CYP108D18 (∼4 μM): (a) oxidised form (black trace) displaying typical ferric heme‐Soret peak (417 nm) and α‐β bands (569 nm and 535 nm). The reduced ferrous form (green trace) CYP108D18 has an absorbance peak at approximately 413 nm. The reduced ferrous CO‐bound CYP108D18 (pink trace) shows the characteristic absorbance peak at 450 nm. (b) Thermal stability analysis of CYP108D18 in comparison with CYP108A1 and CYP108D1. Plot displays the residual P450 heme content/CO‐binding following the incubation at elevated temperatures for 15 and 60 min. The obtained data (*n* = 3) were fitted to a Hill‐type equation (Equation 1) using GraphPad Prism to extract T_50_ values for both the time points. Binding curves for CYP108D18 (1 μM) (*n* = 3) with (c) phenylcyclohexane and (d) phenanthrene. Insets display the spectral shifts observed upon titration of CYP108D18 with each substrate. The absorbance differences between the peak and trough in the spectral shifts were plotted against increasing substrate concentration. The data were fitted to the Morrison tight‐binding equation (Equation 3) using GraphPad Prism allowing the K_D_ values to be obtained.

### 3.3 Thermal Stability of CYP108D18

The thermal stability of CYP108D18 was investigated using CO‐difference spectroscopy. The residual P450 (heme) concentration was quantified after incubating the enzyme at elevated temperatures for 15 or 60 min and compared to the protein retained at 4°C. The resulting data, plotted as residual P450 heme content against temperature, yielded a 15‐min T_50_ values of 47°C ± 1°C for CYP108D18 (Figure 2b). In comparison, the ^15^T_50_ of CYP108D18 was 7°C higher than that of mesophilic enzyme CYP108A1 (40.1°C ± 0.3°C). This improvement in the thermal stability of CY108D18 is moderate in comparison to other CYPs identified from the same hot spring environment (Kundral et al. 2025). Importantly however, the 60‐min T_50_ values of CYP108D18 showed a more significant increase of approximately 14°C (46.0°C ± 0.3°C) when compared to that of CYP108A1 (32.4°C ± 0.5°C) with no loss of activity observed after prolonged incubation at 40°C, in contrast to CYP108A1.

For further comparative analysis, the thermal stability of the related enzyme, CYP108D1, isolated from a *Novosphingobium* sp. strain, was also evaluated. Surprisingly, CYP108D1 exhibited slightly higher ^15^T_50_ and ^60^T_50_ values of 50.0°C ± 0.2°C and 47.5°C ± 0.5°C, respectively compared to those observed for CYP108D18. The relatively lower T_50_ of CYP108D18 may represent an adaptation to the thermal fluctuations characteristic of the hot spring environment in which it was found. In contrast, the greater thermal stability of CYP108D1, previously unreported, could be attributed to its origin in the more thermally stable conditions of Middendorf sediments, a subsurface cretaceous formation located 410 m below ground (Fredrickson et al. 1991). However, further studies are required to clarify the structural features contributing to this enhanced stability.

### 3.4 Binding Studies

A range of aromatic compounds, including aromatic hydrocarbons and monoterpenes, were screened as potential substrates for CYP108D18. Substrate‐induced spectral shifts were monitored using UV‐visible spectroscopy. Characteristic spectral shifts (Type I) occur when a substrate displaces the water molecule coordinated to the heme iron, resulting in a transition of the iron from the low‐spin (~418 nm) to high‐spin (~390 nm) state. Among the tested compounds, the aromatic hydrocarbons phenylcyclohexane and phenanthrene induced the largest spin state shifts (96% ± 2%, and 89% ± 2%, respectively) and bound the tightest (K_D_ = 0.9 ± 0.2, 0.2 ± 0.1 μM, respectively) to CYP108D18 (Table 2, Figure 2c,d, Figure S5). In the reduced state, the substrate‐bound form of CYP108D18 showed an absorbance maximum at 408 nm (Figure S5), a more pronounced shift compared to the substrate‐free reduced form (Figure 2a). Monoterpenes such as *p*‐cymene, (*R*)‐limonene, (*S*)‐limonene and α‐terpineol induced comparatively smaller heme spin‐state shifts and had weaker binding affinities (Table 2).

**TABLE 2:** Heme spin‐state shifts (%) and dissociation constants (K_D_) of tested substrates exhibiting Type I spectral shifts with CYP108D18 (1 μM) (*n* = 3).

Substrate	Heme‐spin shifts (%)	K_D_ (μM)
phenylcyclohexane	96 ± 2	0.9 ± 0.2
phenanthrene	89 ± 2	0.2 ± 0.1
*p*‐cymene	57 ± 6	15 ± 1
α‐terpineol	40 ± 2	28 ± 6
(*R*)‐limonene	61 ± 2	8 ± 3
(*S*)‐limonene	54 ± 4	10 ± 4

### 3.5 Redox Characterisation

Optical spectroelectrochemistry was employed to investigate the redox characteristics of the heme of CYP108D18. The electrochemical reduction of substrate‐free CYP108D18 resulted in a Soret band spectral shift from 417 to 409 nm (Figure 3a). The spectral changes in the heme Soret band (417 nm) and α‐β bands (550 nm) were analysed as a function of applied potentials. A mid‐point potential of −451 mV versus NHE was calculated for the heme in the substrate‐free CYP108D18. The spectroelectrochemical experiments of substrate‐bound (100 μM phenylcyclohexane) CYP108D18 also resulted in a Soret band shift from 394 to 407 nm (Figure 3b). Spectral changes at wavelengths associated with the oxidised, substrate‐bound high spin CYP108D18 (394 nm, 550 nm and 650 nm) were analysed. Data fitting at these wavelengths gave a midpoint potential of −409 mV versus NHE.

**FIGURE 3:**
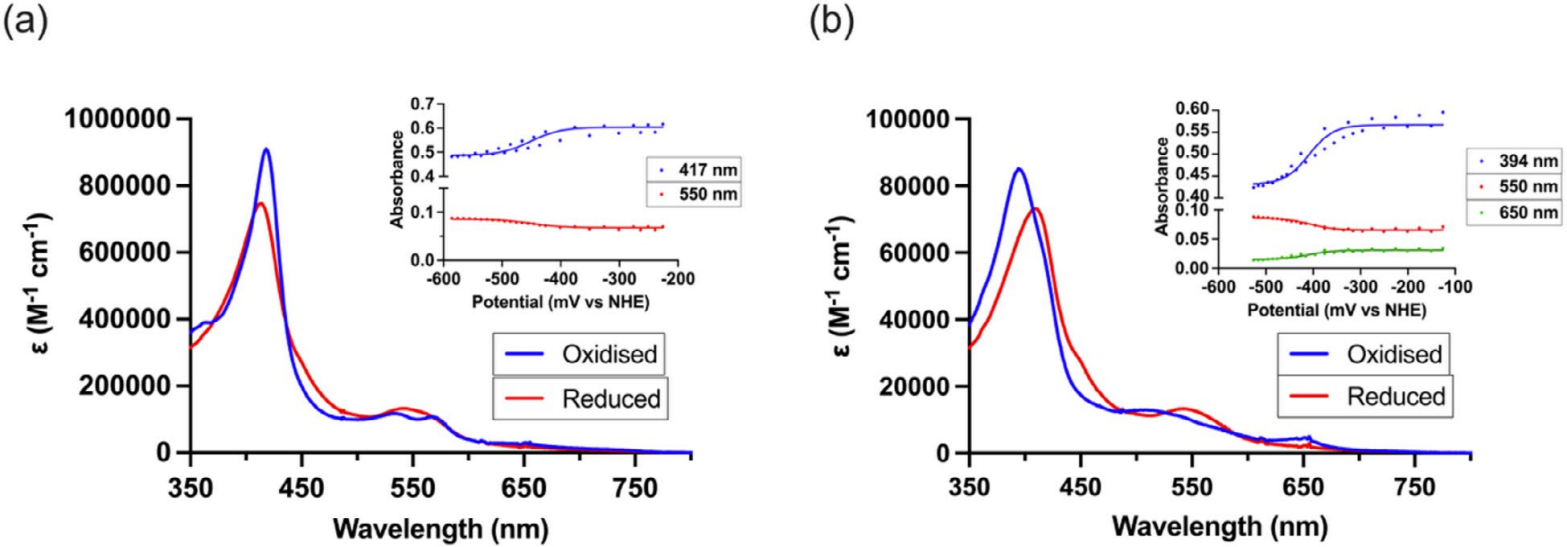
UV‐visible spectra (350–800 nm) of spectroelectrochemical titration of (a) substrate‐free CYP108D18 (30 μM), and (b) substrate‐bound (100 μM phenylcyclohexane) CYP108D18 (30 μM), at 20°C showing both oxidised (blue) and reduced (red) states. Insets Selected single wavelength absorption values as a function of applied potentials.

It is widely accepted that the selective delivery of electrons to substrate‐bound, rather than substrate‐free P450 enzymes may be partially thermodynamically controlled by the redox potential of the heme iron. Substrate binding typically shifts the heme redox potential to a more positive value, thereby making electron transfer from redox partners more thermodynamically favourable. This phenomenon has been well documented in CYP101A1, where the redox potential shifts from −300 mV to −170 mV upon binding D‐camphor (Gunsalus et al. 1973). As a result, the redox partner of CYP101A1, putidaredoxin (−235 mV vs. NHE) becomes capable of more easily reducing the substrate‐bound form of CYP101A1 compared to the substrate‐free form (Sligar and Gunsalus 1976). A similar substrate‐induced shift has also been observed for CYP102A1, where the redox potential shifts from −368 mV to −239 mV upon arachidonate binding (Daff et al. 1997). The unfavourable nature of electron transfer to substrate‐free P450s has been postulated to naturally prevent unproductive electron transfer.

In contrast, while the redox potential measured for substrate‐free CYP108D18 is comparable to those reported for other P450 enzymes (Daff et al. 1997; Hunter et al. 2005), its substrate‐bound form exhibits a relatively small positive change in redox potential. This small redox potential shift is atypical and has not been widely observed except in the related enzyme, CYP108D1 (Bell et al. 2012), where the substrate‐free enzyme exhibited a redox potential of −432 ± 8 mV, shifting to −401 ± 4 mV upon binding phenylcyclohexane. This subtle shift occurs despite clear heme spin‐state changes, as evidenced by a shift in the Soret band. These observations suggest that the limited redox potential change upon substrate binding may be a distinguishing feature of enzymes within the CYP108D subfamily.

### 3.6 NAD(P)H Consumption Assay

In *N. aromaticivorans* , the ArR‐Arx class I electron transfer system efficiently supports the monooxygenase activity of several conspecific P450s, including CYP101B1, CYP101C1, CYP101D1, CYP101D2 and CYP111A2 (Bell et al. 2010). However, previous studies have reported lower activity of this system with CYP108D1 from the same organism (Bell et al. 2012). In this study, the ArR‐Arx system exhibited negligible activity with CYP108D18 for phenylcyclohexane or *p‐*cymene. Therefore, to identify suitable redox partners for CYP108D18, several alternative electron transfer systems were evaluated for their ability to deliver reducing equivalents to CYP108D18. Among all combinations tested, the pairing of spinach ferredoxin (Fdx) and ferredoxin reductase (FdR) resulted in the highest NADPH consumption rate observed, approximately 18 ± 1 μM NADPH min^−1^ per μM of CYP108D18 with phenylcyclohexane as the substrate (Table S1). This is consistent with phenylcyclohexane inducing the highest heme spin‐state shift (> 95%) and exhibiting tight binding to CYP108D18 (K_D_ = 0.9 ± 0.2 μM). In contrast, the same redox partner system showed no detectable activity with *p*‐cymene, with NADPH consumption rates indistinguishable from background levels. The activity of CYP108D18 was also comparatively low when paired with terpredoxin (Tdx), the electron transfer partner of CYP108A1 (Peterson et al. 1992). Other redox systems including putidaredoxin/putidaredoxin reductase (Pdx/PdR) from *P. putida* (Peterson et al. 1990), cindoxin and cymredoxin from *R. globerulus* (Giang et al. 2023; Hawkes et al. 2010) and flavodoxin reductase (FdR) from *E. coli* (Jenkins and Waterman 1994) were also tested in various combinations. However, none of these systems supported any significant catalytic activity of CYP108D18 under the conditions tested.

### 3.7 In Vitro Turnover Assays

Following the identification of spinach ferredoxin (Fdx) and ferredoxin reductase (FdR) as the optimal non‐native redox partners, the catalytic activity of CYP108D18 was successfully reconstituted in vitro (Figure 4). GC–MS analysis of the reaction products revealed a compound that showed a 95% match to *trans*‐4‐phenylcyclohexanol in the GC–MS spectral libraries (Wiley, NIST). The product identity was subsequently confirmed by comparison with an authentic standard of *trans*‐4‐phenylcyclohexanol, which exhibited identical retention time and fragmentation pattern (Figure 4). To further investigate the oxidative capabilities of CYP108D18, all other previously tested substrates for binding were examined; however, none of them produced any detectable product. Similarly, none of the alternative redox partner combinations produced any detectable product (Table S1). In contrast, the spinach Fdx/FdR system supported CYP108D18 activity, which is attributed to its favourable redox potential. Spinach ferredoxin has a midpoint potential of ~−430 mV versus NHE (Cammack et al. 1977; Tagawa and Arnon 1968), which matches with the highly reducing heme redox potential of phenylcyclohexane‐bound CYP108D18 (−409 mV vs. NHE).

**FIGURE 4:**
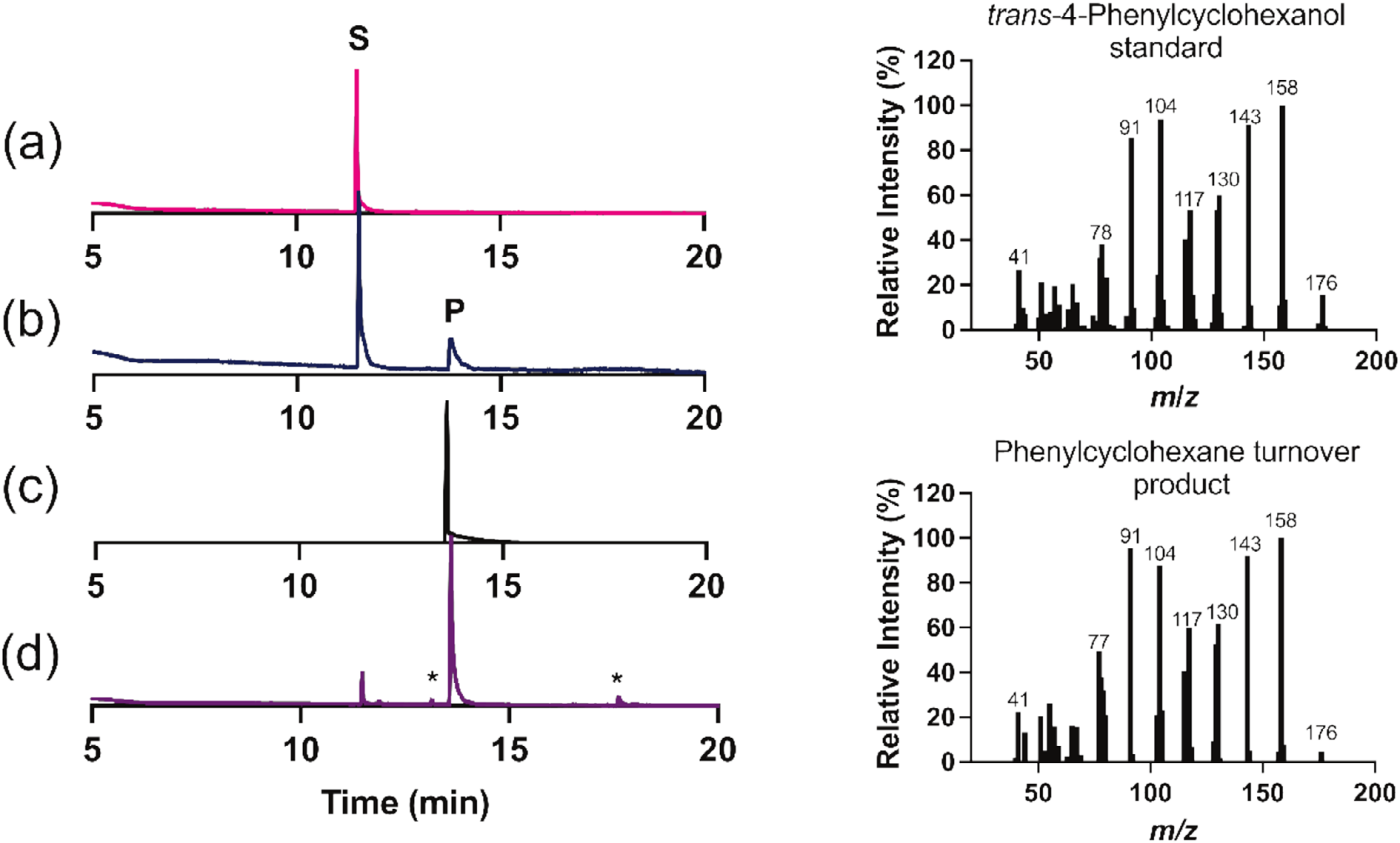
(Left) GC–MS traces of in vitro turnovers of phenylcyclohexane by CYP108D18 (a) Negative control (no CYP108D18) with substrate peak labelled as S. (b) Phenylcyclohexane turnover giving product (P) with a retention time of 13.75 min (*m*/*z* 176). (c) Synthetic standard of *trans*‐4‐phenylcyclohexanol with a retention time of 13.62 min (*m*/*z* 176). (d) Spiked turnover product with the synthetic standard of *trans*‐4‐phenylcyclohexanol giving a single peak of product at 13.6 min. Other peaks marked with stars (*) are various organosilicon contaminants with mass fragmentation patterns unrelated to the substrate or product. (Right) Mass spectrum of *trans*‐4‐phenylcyclohexanol synthetic standard and turnover product.

### 3.8 Overall Structure of CYP108D18

The crystal structure of substrate‐free CYP108D18 was determined at 2.06 Å resolution. The crystallographic R_work_ and R_free_ values refined to 22.45% and 27.10%, respectively. The final model exhibits the characteristic P450 fold and is comprised of the expected 12 α‐helices (A‐L), six 3_10_‐helices and nine β‐sheets (Figure 5a). However, as with several other P450s, some additional helices in addition to those identified as A‐L are also present. In the substrate‐free structure, the asymmetric unit contains one CYP108D18 and 90 water molecules. An additional elongated density in the active site was not long enough to accommodate two separate water molecules but was of an appropriate size and shape for a dioxygen (O_2_) molecule (Figure S6a). All of the polypeptide could be modelled from residues 2 to 436, with the exception of residues 200–209 of the F‐G loop, which were unresolved. This is due to the inherent flexibility in this region which has been observed in many structures of substrate‐free CYP enzymes (Pochapsky et al. 2010). However, compared to CYP108D1 (Bell et al. 2012), eight additional residues (192–199) were observed in the F‐G loop.

**FIGURE 5:**
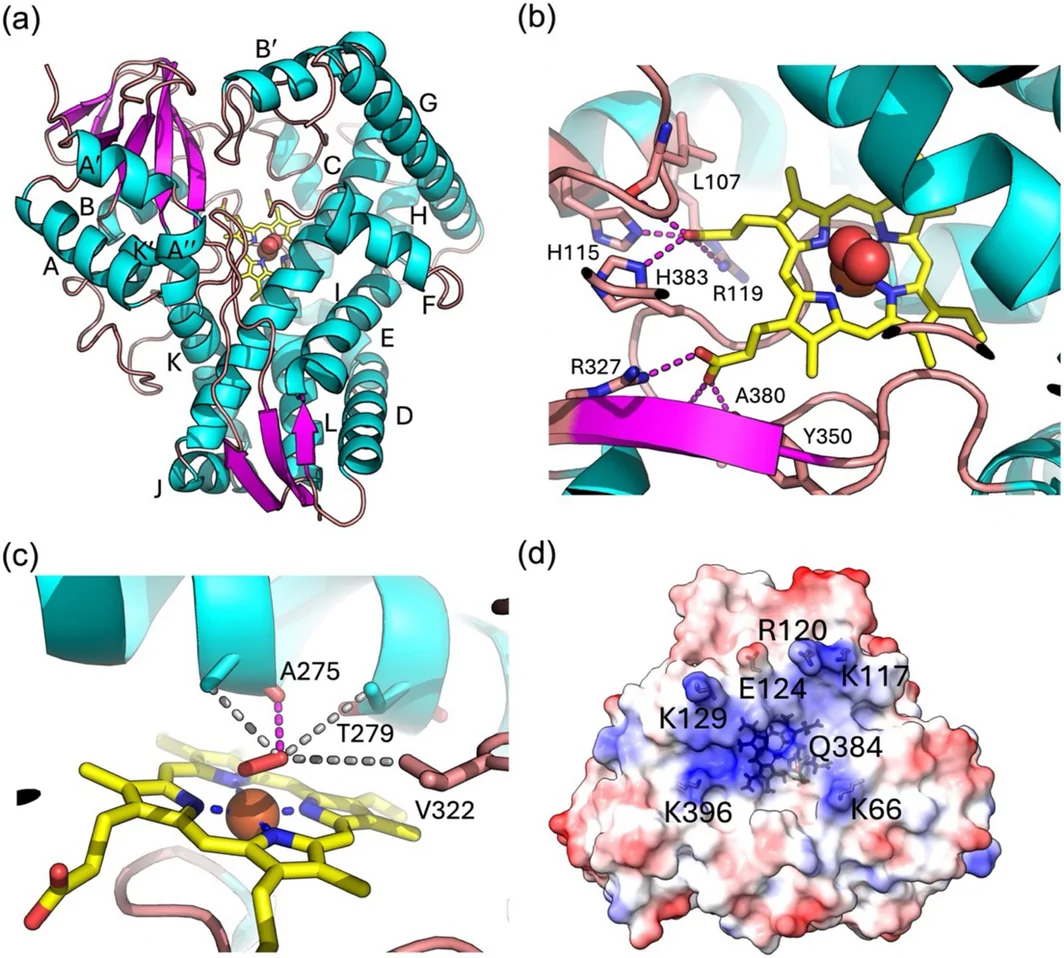
(a) Overall structure of CYP108D18, with α‐helices shown in cyan, β‐strands in magenta and loop regions in brown. The major helices are labelled *A*‐*L*, the heme cofactor is in yellow and the dioxygen is shown as red spheres. (b) Focused view of heme‐propionate interactions with key CYP108D18 residues. Heme‐interacting residues are shown as stick models. (c) Focused view of dioxygen interaction with key CYP108D18 residues. The heme and dioxygen are depicted as stick models and coloured in yellow (carbon atoms) and red, respectively. The Fe centre is shown as an orange sphere. (d) Electrostatic potential surface of the proximal face of CYP108D18. Blue represents positive charge, red negative charge and white neutral.

The propionate groups of the heme in CYP108D18 are stabilised by hydrogen bonds with the main chain nitrogen of Ala380, main chain carbonyl of Leu107 and the sidechains of His115, Arg119, Arg327, Tyr350 and His383 (Figure 5b). A detailed comparison of CYP108D18 with related CYP108 family members, including heme‐binding interactions and overall structural features, is provided in Appendix S1 (Figures S7, S8 and Table S2).

The electron density observed above the heme is tentatively assigned to a dioxygen molecule, modelled to fit the density with an O‐O bond length of 1.2 Å. This dioxygen is positioned 1.8 Å from the heme iron, with an S‐Fe‐O angle of 173.4°, closely resembling the distances and angles observed in the oxy‐complex of CYP101A1 (PDB code: 2A1M; Fe‐O bond length is 1.85 and 1.79 Å, while the S‐Fe‐O angle is 171.3° and 174.2° for molecules A and B, respectively) (Figure S6b) (Nagano and Poulos 2005). In cytochrome P450 enzymes, dioxygen binding occurs only after the reduction of the heme iron from the ferric Fe(III) to the ferrous Fe(II) state. Given that CYP108D18 exhibits a notably low redox potential, spontaneous reduction under crystallisation conditions is unlikely. Therefore, the observed dioxygen possibly results from X‐ray‐induced photoreduction of the ferric heme during X‐ray data collection (Pfanzagl et al. 2020). This suggests that, despite its low redox potential, CYP108D18 can undergo reduction under X‐ray exposure, enabling dioxygen binding and stabilisation within the crystal.

The dioxygen molecule is 2.9 Å from the main‐chain carbonyl of Ala275 and within 4.3 Å of the methyl group on the sidechain of T279, and the sidechains of Ala275 and V322 (Figure 5c). However, no water molecules were observed in the vicinity that would typically participate in the hydrogen‐bonding network stabilising the dioxygen, as seen in another P450‐oxy complex (Nagano and Poulos 2005).

Studies on the proton relay network of P450 enzymes have highlighted the importance of the *I*‐helix ‘acid‐alcohol pair’ residues in stabilising the two water molecules present in the active site and also to facilitate the final proton transfer step for dioxygen cleavage by P450 enzymes (Gerber and Sligar 1992; Imai et al. 1989; Vidakovic et al. 1998). In CYP108D18, Asp278 and Thr279 form the conserved acid‐alcohol pair of the *I*‐helix. The backbone carbonyl of Asp278 forms a hydrogen bond with the backbone amide of Ala282. This orientation is similar to that observed in substrate‐free CYP108A1 (Asp270 and Ser274), CYP108D1 (Asp272 and Thr276) and in the ferrous‐oxy form of CYP101A1 (Asp251 and Asn255) (Bell et al. 2012; Hasemann et al. 1994; Schlichting et al. 2000). In addition, a water molecule (Wat27) is located above the distal *I*‐helix region and forms hydrogen bonds with Asp278, as well as with the carbonyl groups of Thr192 and Ser274 (Figure S9). Another water molecule, Wat12, forms a hydrogen bond with Asp278 and Wat51, which in turn is linked to Wat57 extending into the bulk solvent. This arrangement corresponds to an open conformation, in which Asp278 is part of the proton relay network and promotes opening of the substrate access channel through movements of the *F* and *G* helices.

The ArR‐Arx class I electron transfer system in *N. aromaticivorans* interacts with several CYP101 enzymes in the same organism through both electrostatic and hydrophobic interactions, specifically between positively charged residues on the P450 proximal face and negatively charged residues in Arx. For CYP108D1, a previous study reported reduced but detectable activity with Arx and ArR (Bell et al. 2012). Upon comparison, the proximal faces of CYP108D18 and CYP108D1 show structural similarities. Directly beneath the heme, both enzymes exhibit a less positively charged electrostatic potential on the proximal face compared to the CYP101 enzymes and CYP108A1 (Figure 5d, Figure S10). Similar to CYP108D1, Gln384 of CYP108D18 replaces the arginine residue in CYP101C1, CYP101D1 and CYP101D2, while aligning with Leu356 in CYP101A1 and Met376 in CYP108A1. Glu124 of CYP108D18 replaces the homologous Asp121 in CYP108D1 while aligning with the positively charged lysine residue in the CYP101 enzymes and Asn119 in CYP108A1. These substitutions contribute to a more negatively charged region on the heme‐proximal face of CYP108D18 enzymes and hence have an impact on binding with Arx. Despite the similarities in the heme‐proximal face between CYP108D18 and CYP108D1, no detectable activity of CYP108D18 with Arx and ArR was observed.

Furthermore, distinct structural differences were observed in the proximal loop carrying the Cys ligand in CYP108D18, compared to other P450 enzymes. In CYP108D18, the region from Asp371 to Arg374 protrudes from the proximal surface and extends partially over the heme, a feature not observed in CYP108A1 (Figure S11a). This protrusion is more pronounced in CYP108D1 (Pro365‐Arg368), potentially due to a higher number of proline residues (five in total) within the entire proximal loop (Asp350‐Leu380). In contrast, both CYP108D18 and CYP108A1 contain only two proline residues in the loop. Despite this, CYP108D18 possesses an extended hydrogen‐bonding network within this region, similar to that observed in CYP108D1 (Figure S11b). However, the interaction patterns differ between the two. See Appendix S1 for a detailed comparison.

High‐temperature molecular dynamics (MD) simulations of thermophilic CYP119 have highlighted the proximal loop as a critical structural element contributing to its enhanced thermal stability (Meharenna and Poulos 2010). CYP119, the most thermostable cytochrome P450 known, is derived from the acidothermophilic archaeon *Sulfolobus acidocaldarius* (Wright et al. 1996). It exhibits a melting temperature of ~90°C, and its crystal structure has served as a model for investigating the structural basis of thermostability (McLean et al. 1998; Yano et al. 2000). In CYP119, the proximal loop is stabilised by a cluster of nonpolar residues (Pro306, Leu308, Phe292, Val291 and Tyr26), which anchors the C‐terminal Leu308 to the N‐terminal Tyr26 and disruption of this interaction leads to significant destabilisation. A comparable stabilising feature is observed in CYP108D18, where a cluster comprising Arg374, Leu376, Phe360, Gln359 and Tyr58, though less hydrophobic than that of CYP119, includes a potentially important interaction between Tyr58 and Leu376 that may contribute to the stabilisation of the Cys ligand loop (Figure S11c). Similarly, in CYP108D1, a stabilising interaction between Phe59 and Leu370 is observed. By contrast, mesophilic P450s such as CYP108A1 (His57 and Leu368) and CYP101A1 (Gly68 and Thr348) lack such hydrophobic contacts, which may contribute to their reduced thermostability.

We also determined the crystal structure of CYP108D18 co‐crystallised with phenylcyclohexane. A well‐defined electron density corresponding to phenylcyclohexane was observed above the heme (Figure 6a). This substrate‐bound (SB) form crystallised in the same space group (*C*2_1_) as the substrate‐free enzyme and diffracted to a resolution of 2.25 Å. The structure is similar to that of the substrate‐free form with 12 main α‐helices, six 3_10_‐helices, and nine β‐sheets. In this structure, the loop connecting the *F* and *G* helices also remains partially disordered, similar to that in the substrate‐free form. Alignment of the substrate‐free and substrate‐bound structures shows minimal changes in the overall protein conformation, with an RMSD of only 0.15 Å for all Cα atoms.

**FIGURE 6:**
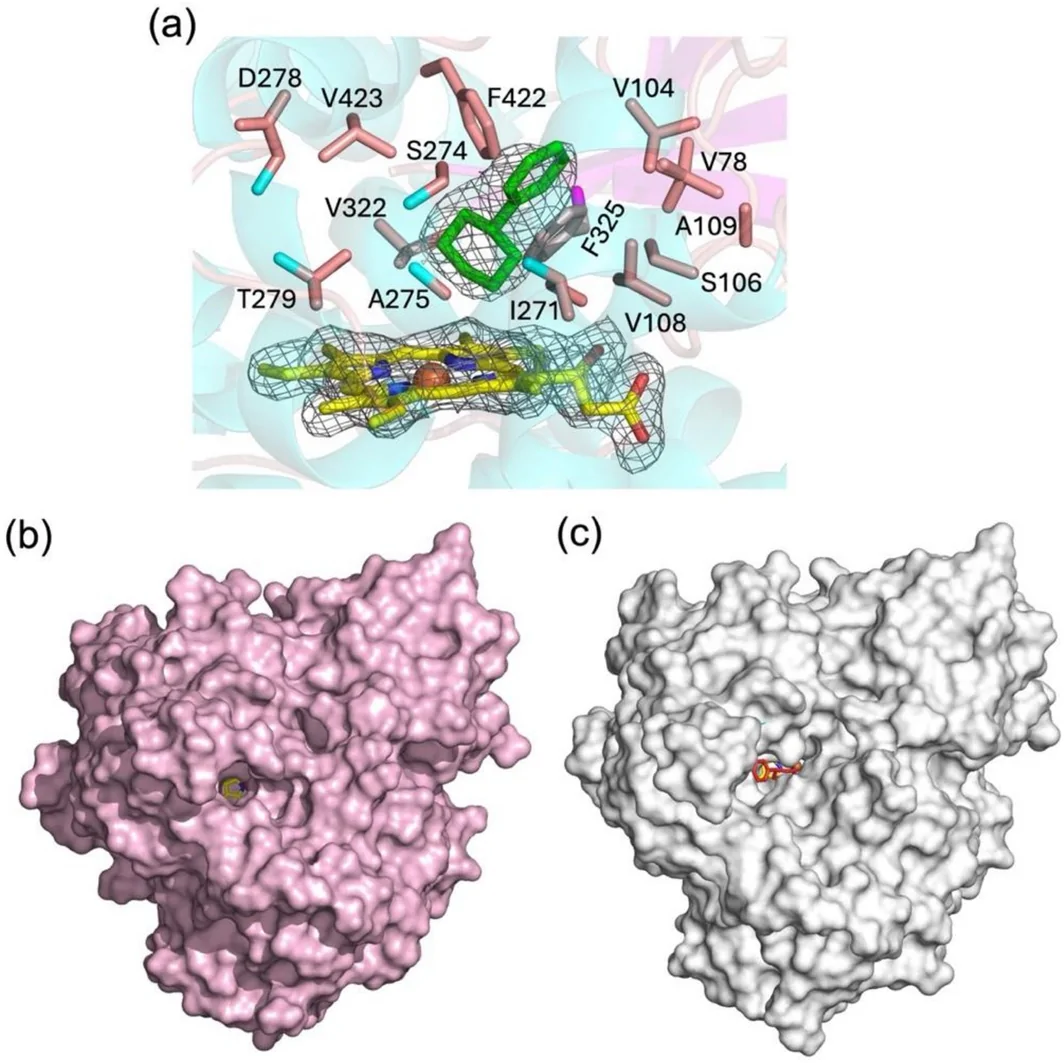
(a) Active‐site view of CYP108D18 co‐crystallised with phenylcyclohexane (green sticks). The 2F_o_‐F_c_ map is shown as a grey mesh contoured at 1.0 *σ* around the substrate and heme. Key residues forming the substrate‐binding pocket are labelled and shown as brown sticks and the heme group is depicted in yellow. Surface representation of CYP108D18 structures (b) substrate‐free and (c) substrate‐bound (phenylcyclohexane) forms. Both structures exhibit an ‘open’ conformation, providing direct access to the heme group (yellow sticks). In panel (c), phenylcyclohexane (red sticks) is visible within the active‐site cavity.

Within the active site (Figure 6a), the C4 position of the aliphatic ring of phenylcyclohexane, proposed as the site of hydroxylation, is positioned 4.5 Å from the heme iron. However, the adjacent C3 position is also located in close proximity, 4.3 Å from the iron centre. For comparison, in CYP108A1 (PDB code: 8EUH), the corresponding carbon atom of α‐terpineol, C7, is 4.2 Å from the iron ion (Gable et al. 2023). Similar to CYP108D1, the access channel of CYP108D18 is more hydrophobic than that of CYP108A1 and has comparable residues including Val79, Thr81, Gly85, Ala89, Leu103 and Phe196 (Table S3). At the bottom of this channel lies the active site, defined by residues Val78, Val104, Ser106, Val108, Ala109, Ile271, Ser274, Ala275, Asp278, Thr279, Val322, Phe325, Phe422 and Val423. In the active site, phenylcyclohexane is stabilised primarily through key nonpolar interactions provided by Ala275, Thr279, Val322, Phe325 and Phe422. Most of these active site residues are conserved between CYP108D18 and CYP108D1, with two notable substitutions: Thr79 and Gln106 in CYP108D1 are replaced by Val78 and Ala109 in CYP108D18. These substitutions increase the hydrophobicity and slightly widen the active site in CYP108D18 compared to CYP108D1. Similarly, in comparison to CYP108A1, the replacement of Glu77, Thr103 and Ser104 with Val78, Val108 and Ala109 in CYP108D18 contributes to a more hydrophobic and spatially open active site environment in CYP108D18 (Table S4). The more hydrophobic and spacious nature of the access channel and active site is consistent with the ability of CYP108D18 to bind bulkier substrates such as phenylcyclohexane, while CYP108A1 favours α‐terpineol.

Previous studies on cytochromes P450 have shown that substrate‐free structures can adopt either open or closed conformations. However, substrate‐bound structures more typically are observed in a closed state (Bell et al. 2008; Madrona et al. 2012). A surface representation of phenylcyclohexane‐bound CYP108D18 reveals an access channel leading to the heme group, with phenylcyclohexane clearly solvent accessible within the open active‐site cavity (Figure 6b,c). Therefore, in this structure there is no closure of the active site as observed in many P450 enzymes. Similar behaviour has been reported for CYP101D2, where camphor binding did not induce major conformational changes (Vohra et al. 2013; Yang et al. 2011). However, further investigation is required to determine whether the open state observed in CYP108D18 corresponds to a functionally relevant conformer associated with the substrate access.

### 3.9 Molecular Docking and Spectroscopic Binding of Terpenes

Despite the relatively strong binding observed for phenylcyclohexane and phenanthrene, these aromatic hydrocarbons are unlikely to represent the physiological substrates of CYP108D18. Given that its host, *Novosphingobium* sp., was identified in a hot‐spring stream surrounded by vegetation, we considered whether CYP108D18 might accommodate plant‐derived hydrophobic compounds potentially present in this environment. To explore this possibility, molecular docking was performed using phenylcyclohexane as a reference ligand and selected representative plant‐derived compounds, including the sesquiterpenes α‐humulene and valencene, the diterpenes isophyllocladene and phytol and the triterpenes β‐sitosterol and moronic acid (Figure S12). Monoterpenes, including *p*‐cymene, α‐terpineol and (*R*)‐limonene, previously tested for binding and activity with CYP108D18, were also included. The corresponding AutoDock Vina scores are provided in Table S5.

The docked pose of phenylcyclohexane closely resembled its experimentally observed binding mode, with the C4 carbon of the cyclohexane ring positioned approximately 4.5 Å from the heme iron, consistent with the crystal structure. The smaller monoterpenes and the larger terpenes could all be accommodated within the open hydrophobic cavity, with a potential oxidation site oriented towards the heme iron. For most compounds, including valencene, phytol, β‐sitosterol, *p*‐cymene, α‐terpineol and (*R*)‐limonene, the carbon atom closest to the heme iron was positioned within 3.6–5.4 Å, consistent with a potentially productive orientation for oxidation. By contrast, α‐humulene, isophyllocladene and moronic acid adopted more distant orientations, with their nearest carbon atoms located up to approximately 7.6 Å from the heme iron, suggesting less favourable positioning for direct oxidation. The ligands were surrounded predominantly by hydrophobic residues, including Val78, Val104, Val108, Ala109, Ile271, Ala275, Phe325, Phe422 and Val423.

These docking results showed that the active‐site cavity of CYP108D18 could accommodate terpenes larger than monoterpenes and prompted their experimental evaluation using spectroscopic binding assays. Heme spin‐state shifts were measured for each compound and compared with that induced by phenylcyclohexane (Table S5). Among the larger terpenes tested, the sesquiterpene valencene produced the largest spin‐state shift (44% ± 9%), although significantly smaller than that observed for phenylcyclohexane (96 ± 2).

Overall, these docking and spectral binding results demonstrate that the active site of CYP108D18 can accommodate structurally diverse hydrophobic compounds, facilitated by the open active‐site channel and large hydrophobic cavity of CYP108D18. Further turnover experiments are required to establish whether valencene and structurally related or functionalised sesquiterpenes are substrates of CYP108D18.

### 3.10 MD Simulations and ONIOM Transition States

A combination of MD simulations and ONIOM calculations were used to assess the likelihood of different hydroxylated products resulting from CYP108D18 catalysed oxidation of phenylcyclohexane. MD simulations of substrate‐bound CYP108D18 in the active oxidant form (Cpd I) were used to identify which hydrogens were well positioned for hydrogen abstraction. Based on the geometric criteria reported by Coleman and co‐workers (Coleman et al. 2022), only C4 and C3 hydrogens were identified in catalytically relevant positions, however, with only < 1% of total snapshots. Specifically, the distribution of H‐O distances, C‐H‐O angles and Fe‐O‐H angles showed that the C4 hydrogen *trans* to phenyl (i.e., Hδeq) and the C3 hydrogen Hγax most often achieve conformations similar to transition state structures calculated with quantum mechanics (QM) methods that are unencumbered by the protein environment (Figure S13).

Furthermore, throughout the MD simulations, CYP108D18 remained in an open conformation: the substrate‐binding pocket was open to the bulk solvent, but the heme was shielded from the water molecules while the substrate remained bound (Figures S14 and S15). This bulky substrate, complementary to the access channel, can act as a form of lid and remove the need for the enzyme to adopt a closed conformation to facilitate catalysis.

The substrate conformations could be clustered into two major groups defined largely by whether the benzylic hydrogen was oriented towards or away from the *I*‐helix (Figure S16). Both Hδeq and Hγax were close to the iron‐oxo in the largest cluster. A representative snapshot was selected, and the geometry of the reactant complex was optimised, followed by TS structures for abstraction of each hydrogen, using the ONIOM method (Figure S17). The barrier for abstraction of Hδeq was 4 kcal mol^−1^ lower in energy. Therefore, the most likely hydroxylated product would be *trans*‐4‐phenylcyclohexanol. An energy decomposition analysis revealed that while the QM component of the Hγax TS (23.9 kcal mol^−1^) was lower in energy compared to Hδeq (25.9 kcal mol^−1^), the main difference in the MM layer arose from steric clashes between the substrate and residues S274 and V322, ultimately favouring abstraction of Hδeq (Figure S18) as was indicated experimentally.

## 4 Conclusion

In this study, we identified and characterised CYP108D18, the first CYP108 enzyme identified from a metagenome‐assembled genome derived from a geothermal environment. CYP108D18 exhibited moderate thermostability and shared high similarity in both sequence and stability with CYP108D1, which was previously isolated from a subsurface Cretaceous formation in North America, although the thermostability of CYP108D1 had not been fully recognised until now. Despite the vast geographic separation of their source habitats, the close similarity in sequence and stability highlights an unexpected conservation within this enzyme subfamily across distant habitats. CYP108D18 displays a very low redox potential, which does not shift as much as expected upon substrate binding, at least not within the range typically required for efficient electron transfer from the surrogate [2Fe‐2S] ferredoxin partners to the P450 heme. The enzyme binds aromatic substrates and oxidises phenylcyclohexane, forming *trans*‐4‐phenylcyclohexanol as the product. In silico docking and spectroscopic‐binding studies further indicate that CYP108D18 may also interact with larger plant‐derived terpenes, such as sesquiterpenes, highlighting the need to investigate their catalytic turnover.

CYP108D18 represents the third structurally characterised member of the CYP108 family and the first CYP108D enzyme with a structure determined in complex with a substrate. Crystal structures of both the substrate‐free and substrate‐bound forms of CYP108D18 reveal canonical P450 folds. Interestingly, molecular oxygen was observed bound in the substrate‐free structure at 38% occupancy. The bound species is best described as an oxy‐complex with resonance contributions from Fe(III)‐(O_2_^−^) <‐> Fe(II)‐O_2_. This observation is most plausibly attributed to X‐ray‐induced reduction during data collection, leading to heme reduction and subsequent oxygen binding. In the substrate‐bound structure, the C4 position of the hydroxylated phenylcyclohexane product was positioned close to the heme iron. However, no major structural differences were observed between the two CYP108D18 forms and other CYP108s that might explain the unusually low redox potentials. Interestingly, the CYP108D18 enzyme does not exhibit the characteristic open‐to‐closed conformational transition typically associated with substrate binding in many other P450 enzymes. This lack of structural change upon substrate binding may, in part, contribute to the unusually low redox potentials observed in CYP108D18 as well as in CYP108D1. One hypothesis is that the heme remains relatively solvent‐exposed in these enzymes due to the absence of a significant open‐to‐closed transition upon substrate binding. Greater heme exposure to water has been empirically linked to increasingly negative Fe^III/II^ redox potentials in heme proteins, which could explain the unusually low redox potentials of CYP108D18 and CYP108D1 (Stellwagen 1978). However, further experimental validation will be crucial to test this mechanistic hypothesis and establish the physiological function of CYP108D18.

## Author Contributions

**Simran Kundral:** investigation, writing – review and editing, methodology, writing – original draft. **Peter D. Giang:** methodology, writing – review and editing. **Alicia M. Kirk:** methodology, writing – review and editing. **Julia B. Buczynski:** methodology. **Kasun S. A. Arachchige:** methodology, investigation. **Sunil K. Khare:** writing – review and editing. **Paul V. Bernhardt:** methodology, writing – review and editing. **Paul N. Evans:** methodology, writing – review and editing, conceptualization. **Luke W. Guddat:** methodology, investigation, writing – review and editing. **Stephen G. Bell:** writing – review and editing, conceptualization, resources. **James J. De Voss:** conceptualization, writing – review and editing, resources, supervision.

## Funding

This work was supported by the Australian Research Council, DP210103970.

## Conflicts of Interest

The authors declare no conflicts of interest.

## Supporting information


**Figure S1:** Overlay of crystal structure (yellow) and AlphaFold3 predicted structure (purple) compared to equilibrated structure (blue) showing the change in the conformation of the *F*‐*G* region. Substrate and heme shown in green. Two rotated views shown.
**Figure S2:** The amino acid sequence alignment of characterised enzymes from families CYP108 and CYP101 was performed using Clustal Omega web server and visualised by ESpript 3.0 software.
**Figure S3:** UV–visible spectra (250–700 nm) of purified CYP108D18. The observed Reinheitszahl (RZ, A_417_/A_280_) is 1.4, indicative of pure P450.
**Figure S4:** SDS‐PAGE (NuPAGE 4%–12%) analysis of CYP108D18 protein purified using Ni‐NTA affinity chromatography. The observed band corresponds to CYP108D18 (48 kDa) containing additional N‐terminal residues from the expression vector (His_6_‐tag and thrombin cleavage site). Masses of the protein ladder (ThermoFisher, Novex Sharp Pre‐Stained Protein Standard) are indicated.
**Figure S5:** UV‐visible spectra (300–700 nm) of purified CYP108D18 in its oxidised form (black), in the presence of substrates phenylcyclohexane (orange) and phenanthrene (blue), and substrate‐bound (phenylcyclohexane) reduced state (green).
**Figure S6:** (a) Fo‐Fc omit map in green mesh for O_2_ in the substrate‐free structure of CYP108D18. (b) Superposition of CYP108D18 with the oxy‐ferrous complex of CYP101A1. The heme and putative dioxygen ligand of CYP108D18 are shown in yellow and red, respectively, while the heme and dioxygen ligand of molecule A of CYP101A1 (PDB: 2A1M) are shown in cyan. The proximal cysteine residues from both structures are also displayed.
**Figure S7:** Secondary structure comparison between (a) CYP108D18 (wheat), (b) CYP108D1 (PDB: 3TKT, blue) and (c) CYP108A1 (PDB code: 1CPT, cyan). The β‐sheets β1‐5, β4 (β4‐1, β4‐2) and β5 (β5‐1, β5‐2) present in CYP108A1 are replaced by loops in CYP108D18 and CYP108D1 (highlighted in red).
**Figure S8:** Secondary structure comparison of (a) CYP108D18 (wheat), (b) CYP108D1 (PDB: 3TKT, blue), and (c) CYP108A1 (PDB: 1CPT, cyan). The *B′* helix and *E* helix are highlighted in red and green, respectively, across all structures.
**Figure S9:** A proposed short proton delivery pathway in CYP108D18. A chain of water molecules extends from the protein surface to the OD2 atom of Asp278, which is hydrogen‐bonded to a single water molecule (Wat27) located above the *I*‐helix groove.
**Figure S10:** Electrostatic potential of the proximal face of (a) CYP101A1, (b) CYP101C1, (c) CYP101D1, (d) CYP101D2, (e) CYP108A1 and (f) CYP108D1.
**Figure S11:** (a) Focused view of the orientation of the region from residues Asp371 to Arg374, the proximal loop and heme (yellow) of CYP108D18 (brown), compared to CYP108D1 (magenta) and CYP108A1 (green). (b) Orientation of the Asp371‐Arg374 residues in CYP108D18, showing hydrogen bonds as black dashed lines and bridging water molecule in orange. (c) Structural model of the loop in CYP108D18, highlighting the interaction between Tyr58 and Leu376, which may tether the N‐terminal and C‐terminal regions, contributing to structure stabilisation.
**Figure S12:** Docking poses of selected ligands in the active site of CYP108D18. Phenylcyclohexane (a), monoterpenes (b–d), sesquiterpenes (e and f), diterpenes (g and h), and triterpenes (i and j) are shown in salmon, blue, magenta, green and cyan, respectively. Key active‐site residues and the heme group are shown as grey sticks. Yellow dashed lines indicate the distances (Å) between the heme iron and the nearest ligand carbon atom.
**Figure S13:** Distribution of C‐H‐O and Fe‐O‐H angles (°) and H‐O distances (Å) for cyclohexyl hydrogens during MD simulations of phenylcyclohexane bound CYP108D‐CPDI. Black points mark QM TS geometries obtained (Coleman et al. 2022). Margins of ±20° are outlined with dashed lines. Conformations within these margins and with H‐O < 4 Å were considered catalytically relevant and the number of snapshots meeting these criteria are annotated in red. Note a total of 36,150 snapshots were analysed.
**Figure S14:** The *F*‐*G* loop (highlighted in yellow) showed the largest RMSF. (a/b) Rotated views showing overlay of snapshots sampled evenly along the MD simulations. Bound substrate (green), heme (dark blue), protein (blue), residues 196‐213 (yellow). (c) RMSF per residue calculated for the MD simulations. The region of largest fluctuations is from the *F*‐*G* loop inclusive of residues 196‐213. Fluctuations do not contribute to a closed conformation.
**Figure S15:** CYP108D18 remained in an open conformation. (a) RMSD (Å) of the protein during MD simulations and RMSD based clustering assignment over time (ns). Cumulative time shown for the portions of the three simulations where the substrate remains bound. Run 2 can be seen to settle into the stable cluster 0. (b) Overlay of crystal structure (yellow) with representative snapshot from the MD simulations based on the largest cluster (cluster 0) determined through protein RMSD (blue). Water molecules within 6 Å of the bound phenylcyclohexane substrate, sampled across the MD trajectory, shown to illustrate how the binding pocket remains open to the bulk solvent. (c) Overlay of snapshots taken across the MD trajectory showing how the phenylcyclohexane substrate blocks water from the heme while it remains bound. Water shown with red spheres. Heme and substrate in blue.
**Figure S16:** Breakdown of the clusters identified for the MD simulations of CYP108D18 with phenylcyclohexane bound. Clusters 4–16 make up the remaining 5%. Note in c2 the substrate is further from the heme in general. Distribution of C‐H‐O and Fe‐O‐H angles (°) and H‐O distances (Å) for cyclohexyl hydrogens during MD simulations of phenylcyclohexane bound CYP108D‐CPDI. Black points mark QM TS geometries obtained from previous work (Coleman et al. 2022). Margins of ±20° are outlined with dashed lines. Conformations within these margins and with H‐O < 4 Å were considered catalytically relevant and the number of snapshots meeting these criteria are annotated in red. Note a total of 36,150 snapshots were analysed.
**Figure S17:** Structures of (a) reactant complex, (b) TS of Hγax abstraction and (c) TS of Hδeq abstraction calculated at the ONIOM (DFT:AMBER) level of theory. QM region coloured green. TS bonds shown using black dashed lines. Key bond distances (Å) and angles (°) are annotated. Energies relative to the reactive complex given in kcal mol^−1^ with zero‐point‐energy corrections.
**Figure S18:** Energy decomposition comparison between TS for Hγax and Hδeq abstraction. The largest differences indicating a larger energetic cost in TS Hγax correspond to removal of interactions for residues S274, V322, and the substrate (PCH 438).
**Table S1:** NAD(P)H consumption rate (μM min^−1^ per μM CYP108D18) of CYP108D18 with different redox partners in the presence of substrate (1 mM). Background rate of NAD(P)H consumption due to redox partners were measured in the absence of P450 and subtracted from calculated rates (*n* = 2). ^a^NADH was used; ND indicates not detected. The formation of hydroxylated products was confirmed by GC–MS analysis. ‘Y’ indicates detectable product formation, while ‘N’ denotes no product detected.
**Table S2:** Comparison of heme propionate interactions with key residues in CYP108D18, CYP108D1 and CYP108A1.
**Table S3:** Comparison of the access channel residues of CYP108D18, CYP108D1 and CYP108A1.
**Table S4:** Comparison of the active site residues of CYP108D18, CYP108D1 and CYP108A1.
**Table S5:** AutoDock Vina docking scores of phenylcyclohexane and selected plant‐derived hydrophobic compounds within the active site of CYP108D18.

## Data Availability

The data that support the findings of this study are openly available in European Nucleotide Archive (ENA) at https://www.ebi.ac.uk/ena/browser/home, reference number PRJEB88793.
