# Supplementary material for: Structural and Functional Characterisation of a Metagenome‐Derived CYP108D18 Reveals an Unusual Dioxygen‐Bound State and Selective Hydrocarbon Hydroxylation
Source: Microb Biotechnol. 2026 Sep 30;19(10):e70451. doi: 10.1111/1751-7915.70451 (PMC13627977; doi:10.1111/1751-7915.70451)
Supplement: Supplementary file 1 [file MBT2-19-e70451-s001.docx]

**Supporting Information**

***Structural and Functional Characterisation of a Metagenome-Derived CYP108D18 Reveals an Unusual Dioxygen-Bound State and Selective Hydrocarbon Hydroxylation***

*Simran Kundral ^a b c^, Peter D. Giang ^b^, Alicia M. Kirk ^b d^, Julia B. Buczynski ^b^, Kasun S. A. Arachchige ^e^, Sunil K. Khare ^c f^, Paul V. Bernhardt ^b^, Paul N. Evans ^g^,* *Luke W. Guddat ^b^, Stephen G. Bell ^h^, James J. De Voss ^b*^*

*^a^ The University of Queensland - Indian Institute of Technology Delhi (UQIITD) Research Academy, Hauz Khas, New Delhi 110016, India*

*^b^ School of Chemistry and Molecular Biosciences, The University of Queensland, St Lucia, QLD 4072, Australia*

*^c^ Enzyme and Microbial Biochemistry Laboratory, Department of Chemistry, Indian Institute of Technology Delhi, Hauz Khas, New Delhi 110016, India*

*^d^ Scuola Normale Superiore, Piazza dei Cavalieri 7, 56126 Pisa, Italy*

*^e^ Centre for Microscopy and Microanalysis, The University of Queensland, St. Lucia, QLD 4072, Australia*

*^f^ Department of Biological Sciences, Indian Institute of Science Education and Research Kolkata, Mohanpur, West Bengal 741246, India*

*^g^ Australian Centre for Ecogenomics, School of Chemistry and Molecular Biosciences, The University of Queensland, St Lucia, QLD 4072, Australia*

*^h^ School of Physics, Chemistry and Earth Sciences, Adelaide University, Adelaide, South Australia 5005, Australia*

Table of Contents

[Amino-acid sequence of CYP108D18	3](#_Toc238679072)

[Experimental section	4](#_Toc238679073)

[Spectroelectrochemistry - inorganic mediator structures	4](#_Toc238679074)

[Computational details of molecular dynamics simulations	4](#_Toc238679075)

[Results and Discussion	7](#_Toc238679076)

[Amino acid sequence alignment	7](#_Toc238679077)

[Spectroscopic characterisation	8](#_Toc238679078)

[CYP108D18 SDS-PAGE analysis	9](#_Toc238679079)

[Binding studies	9](#_Toc238679081)

[Structural characterisation	10](#_Toc238679082)

[Docking studies	16](#_Toc238679083)

[Molecular dynamics simulations and ONIOM transition states	17](#_Toc238679084)

[References	25](#_Toc238679085)

# Amino-acid sequence of CYP108D18

MGSSHHHHHHSSGLVPRGSHMMTGPVASPADALAVIDPKSYADWTAALDLFDRLRAETPVVRIESPTNDHEPFWLVTKYDDVMRISKDNATFLNAPRSVVFTNRGGEALARQVTAQQGDESPNLVSSLVAMDAPKHPKMRRLTQEWFMPKNLRSLESRVRELARATVERFIAAGGDSGRADFVTHVAAPYPLHVVMQILGVPEEDEPKMLFLTQQMFGGQDEDLSKSGMANMTPEMISQIVFGAVAEFETYFEGLAAQRRANPTDDVASILATAEIDGEPISARDRAGYYIITASAGHDTTSASTAGAMLALARDPEQFAKVKADRSLLANIVEEAIRWTTPVQHFMRMANEDVELSGVKIAKGDWIMINYVAANHDPAQFDNPRKFDVTRDQTRHLAFGAGAHQCLGLHLARLEMKLLFEALLDRIESIELAGEPRRAKSTFVGGLKTLPLRFTAS

**Note:** The protein was expressed with an N-terminal His_6_-affinity tag followed by a thrombin cleavage site (SSGLVPRGSH). Residues originating from the expression vector are highlighted in green.

# Experimental section

## Spectroelectrochemistry - inorganic mediator structures

**Scheme S1** Inorganic redox mediator complexes used in this work and their redox potentials (mV vs. NHE at pH 7) (Bernhardt et al., 2006).

## Computational details of molecular dynamics simulations

Parameters for the thiolate coordinated heme (Compound I) were taken from Cheatham and coworkers (Shahrokh *et al.*, 2012). Parameters for PCH were generated with AmberTools25 using antechamber with AM1-BCC charges and the General Amber Force Field (gaff2). The Amber 14 force field with Stony Brook modifications (ff14SB) was employed for the protein. The system was solvated in a 10 Å box of TIP3P water and the charge neutralised with 12 Na^+^ ions. Periodic boundary conditions were employed and the particle-mesh-Ewald method for long range electrostatic effects, while a 10 Å cut-off was employed for Lennard-Jones and electrostatic interactions. The SHAKE algorithm was employed to constrain the water molecules (Ryckaert *et al.*, 1977; Miyamoto and Kollman, 1992). Positional restraints of 300 kcal mol^-1^ Å^-2^ were placed on the protein, heme, and PCH. The energy of the system was minimised. The system was heated to 300 K over 300 ps under constant-volume and a time step of 1 fs. To control the temperature, the Andersen-like coupling scheme was used. The restraints were then reduced to only the alpha carbon of residues 1-180, 243-436 along with the heme nitrogens and PCH. The density of the system was equilibrated for 2 ns and a time step 2 fs under constant pressure of 1 atm using the Berendsen barostat and relaxation time of 5 ps. Maintaining the restraints, the system was then equilibrated under constant volume for 2ns. Over an additional 6 ns, NMR restraints of 10 kcal mol^-1^ Å^-2^ were gradually applied to decrease the distances between the following residues: Gln193 and Asp278 and His277; maintaining His277 and Ala167; Met175 and Val180. All restraints were then released and the system equilibrated for a further 2 ns. The substrate remained bound. Triplicate production runs were performed for 500 ns or until the substrate moved significant distance from the heme. The substrate remained bound for the entire duration in one replicate while moved into catalytically irrelevant conformation at 75 ns and 148 ns for the remaining replicates respectively. Snapshots were recorded every 10000 steps (every 20 ps).

For each snapshot, the positions of the PCH cyclohexyl hydrogens relative to Cpd I were analysed in terms of H-O bond distances, C-H-O angles, and H-O-Fe angles using *cpptraj* package (Roe and Cheatham, 2013). These key geometries were compared to those in transition state (TS) structures of hydrogen abstraction calculated using density functional theory (DFT) and a truncated heme model with 4-cyclohexylbenzoic acid published by Coleman and co-workers (Coleman *et al.*, 2022). The DFT TS geometries for 4-cyclohexylbenzoic acid were assumed to be similar and analogous for these purposes to those of phenylcyclohexane given the carboxylic acid functional group is remote from the site of reactivity and not sterically determinant in the geometries. The number of snapshots that were within 20° of the above angles, with a H-O distance < 4 Å were determined and used to predict the positional likelihood of abstraction of each hydrogen. Clustering was performed using the hierarchal method in *cpptraj* (Roe and Cheatham, 2013) for a total of 16 clusters with epsilon 100.0 based on the above geometric parameters.

|  |  |
| --- | --- |
|  |  |

**Figure S1** Overlay of crystal structure (yellow) and AlphaFold3 predicted structure (purple) compared to equilibrated structure (blue) showing the change in the conformation of the F-G region. Substrate and heme shown in green. Two rotated views shown.

# Results and Discussion

## Amino acid sequence alignment

**Figure S2** The amino acid sequence alignment of characterised enzymes from families CYP108 and CYP101 was performed using Clustal Omega web server and visualised by ESpript 3.0 software.

## Spectroscopic characterisation

**Figure S3** UV-visible spectra (250–700 nm) of purified CYP108D18. The observed Reinheitszahl (RZ, A_417_/A_280_) is 1.4, indicative of pure P450.

## CYP108D18 SDS-PAGE analysis

**Figure S4** SDS-PAGE (NuPAGE 4–12%) analysis of CYP108D18 protein purified using Ni-NTA affinity chromatography. The observed band corresponds to CYP108D18 (48 kDa) containing additional N-terminal residues from the expression vector (His_6_-tag and thrombin cleavage site). Masses of the protein ladder (ThermoFisher, Novex Sharp Pre-Stained Protein Standard) are indicated.

## Binding studies

**Figure S5** UV-visible spectra (300 − 700 nm) of purified CYP108D18 in its oxidised form (black), in the presence of substrates phenylcyclohexane (orange) and phenanthrene (blue), and substrate-bound (phenylcyclohexane) reduced state (green).

## Structural characterisation

**Figure S6 (a)** Fo-Fc omit map in green mesh for O_2_ in the substrate-free structure of CYP108D18. **(b)** Superposition of CYP108D18 with the oxy-ferrous complex of CYP101A1. The heme and putative dioxygen ligand of CYP108D18 are shown in yellow and red, respectively, while the heme and dioxygen ligand of molecule A of CYP101A1 (PDB: 2A1M) are shown in cyan. The proximal cysteine residues from both structures are also displayed.

Superposition of CYP108D18 with CYP108D1 (PDB code: 3TKT) resulted in a RMSD of 0.54 Å for 412 Cα atoms (Bell *et al.*, 2012). Superposition with CYP108A1 (PDB code: 1CPT) resulted in a higher RMSD of 1.16 Å across 403 Cα atoms. Similar to CYP108D1, CYP108D18 possesses shorter and fewer β-sheets compared to CYP108A1 (**Figure S7**). For instance, residues 252-261 between the *H* and *I* helices form a loop in CYP108D18, replacing two small β-sheets present in CYP108A1. Furthermore, CYP108D18 features a larger *B'* helix and a more complete *E* helix relative to both CYP108D1 and CYP108A1 (**Figure S8**).

A similar set of interactions are observed in CYP108A1 and CYP108D1, except Ala380 is replaced by Trp372 in CYP108A1 (**Table S2**). Although the heme-ligand environment is known to modulate the P450 spin state and redox potential (Loew and Harris, 2000), these conserved interactions do not account for the lower reduction potential of the CYP108D enzymes. Therefore, detailed computational studies will be required to determine whether such subtle variations in heme-protein interactions or other factors contribute to their low redox potentials.

**Figure S7** Secondary structure comparison between **(a)** CYP108D18 (wheat), **(b)** CYP108D1 (PDB: 3TKT, blue), and **(c)** CYP108A1 (PDB code: 1CPT, cyan). The β-sheets β1-5, β4 (β4-1, β4-2) and β5 (β5-1, β5-2) present in CYP108A1 are replaced by loops in CYP108D18 and CYP108D1 (highlighted in red).

**Figure S8** Secondary structure comparison of **(a)** CYP108D18 (wheat), **(b)** CYP108D1 (PDB: 3TKT, blue), and **(c)** CYP108A1 (PDB: 1CPT, cyan). The B' helix and E helix are highlighted in red and green, respectively, across all structures.

**Figure S9** A proposed short proton delivery pathway in CYP108D18. A chain of water molecules extends from the protein surface to the OD2 atom of Asp278, which is hydrogen-bonded to a single water molecule (Wat27) located above the I-helix groove.

**Figure S10** Electrostatic potential of the proximal face of **(a)** CYP101A1, **(b)** CYP101C1, **(c)** CYP101D1, **(d)** CYP101D2, **(e)** CYP108A1 and **(f)** CYP108D1.

**Figure S11** **(a)** Focused view of the orientation of the region from residues Asp371 to Arg374, the proximal loop and heme (yellow) of CYP108D18 (brown), compared to CYP108D1 (magenta) and CYP108A1 (green). **(b)** Orientation of the Asp371-Arg374 residues in CYP108D18, showing hydrogen bonds as black dashed lines and bridging water molecule in orange. **(c)** Structural model of the loop in CYP108D18, highlighting the interaction between Tyr58 and Leu376, which may tether the N-terminal and C-terminal regions, contributing to structure stabilisation.

In CYP108D1, loop residues form hydrogen bonds with both the *K* and *L* helices, whereas in CYP108D18, interactions are limited to the *K* helix. Specifically, the sidechain amide group of Gln372 in CYP108D18 forms hydrogen bonds with the sidechain of Glu314 (*K* helix) and the main chains of Arg370 and Asp367. In CYP108D1, the main-chain nitrogen and sidechain of Asn367 interact with Glu307 (*K* helix) and Arg386 (*L* helix), respectively. Additionally, in CYP108D18, the sidechain and main-chain carbonyl of Arg374 form hydrogen bonds with the sidechain of Gln359 and the hydroxyl group of Tyr58 in the N-terminal region, respectively. A water molecule also coordinates with the NH_2_ group of Arg374. This protruding loop has previously been associated with slow electron transfer in CYP108D1 (Bell *et al.*, 2012). To realign the loop in CYP108D1, a mutation substituting Pro365 with valine was tested; however, it did not improve ferredoxin binding, suggesting that loop repositioning alone may not be sufficient. Therefore, further studies are required to fully elucidate the functional impact of this structural distortion in both CYP108D1 and CYP108D18.

## Docking studies

**Figure S12** Docking poses of selected ligands in the active site of CYP108D18. Phenylcyclohexane **(a)**, monoterpenes **(b, c & d)**, sesquiterpenes **(e & f)**, diterpenes **(g & h)**, and triterpenes **(i & j)** are shown in salmon, blue, magenta, green and cyan, respectively. Key active-site residues and the heme group are shown as grey sticks. Yellow dashed lines indicate the distances (Å) between the heme iron and the nearest ligand carbon atom.

## Molecular dynamics simulations and ONIOM transition states

**Figure S13** Distribution of C-H-O and Fe-O-H angles (°) and H-O distances (Å) for cyclohexyl hydrogens during MD simulations of phenylcyclohexane bound CYP108D-CPDI. Black points mark QM TS geometries obtained (Coleman et al., 2022). Margins of ± 20° are outlined with dashed lines. Conformations within these margins and with H-O <4 Å were considered catalytically relevant and the number of snapshots meeting these criteria are annotated in red. Note a total of 36150 snapshots were analysed.

**Figure S14** The F-G loop (highlighted in yellow) showed the largest RMSF. **(a/b)** Rotated views showing overlay of snapshots sampled evenly along the MD simulations. Bound substrate (green), heme (dark blue), protein (blue), residues 196-213 (yellow). **(c)** RMSF per residue calculated for the MD simulations. The region of largest fluctuations is from the F-G loop inclusive of residues 196-213. Fluctuations do not contribute to a closed conformation.

**Figure S15** CYP108D18 remained in an open conformation. **(a)** RMSD (Å) of the protein during MD simulations and RMSD based clustering assignment over time (ns). Cumulative time shown for the portions of the three simulations where the substrate remains bound. Run 2 can be seen to settle into the stable cluster 0. **(b)** Overlay of crystal structure (yellow) with representative snapshot from the MD simulations based on the largest cluster (cluster 0) determined through protein RMSD (blue). Water molecules within 6 Å of the bound phenylcyclohexane substrate, sampled across the MD trajectory, shown to illustrate how the binding pocket remains open to the bulk solvent. **(c)** Overlay of snapshots taken across the MD trajectory showing how the phenylcyclohexane substrate blocks water from the heme while it remains bound. Water shown with red spheres. Heme and substrate in blue.

|   c0 - 41% |   c1 - 29% |
| --- | --- |
|   c2 - 17% |   c3 - 8% |

**Figure S16** Breakdown of the clusters identified for the MD simulations of CYP108D18 with phenylcyclohexane bound. Clusters 4-16 make up the remaining 5%. Note in c2 the substrate is further from the heme in general. Distribution of C-H-O and Fe-O-H angles (°) and H-O distances (Å) for cyclohexyl hydrogens during MD simulations of phenylcyclohexane bound CYP108D-CPDI. Black points mark QM TS geometries obtained from previous work (Coleman et al., 2022). Margins of ± 20° are outlined with dashed lines. Conformations within these margins and with H-O < 4 Å were considered catalytically relevant and the number of snapshots meeting these criteria are annotated in red. Note a total of 36150 snapshots were analysed.

**Figure S17** Structures of **a)** reactant complex, **b)** TS of Hγax abstraction, and **c)** TS of Hδeq abstraction calculated at the ONIOM (DFT:AMBER) level of theory. QM region coloured green. TS bonds shown using black dashed lines. Key bond distances (Å) and angles (°) are annotated. Energies relative to the reactive complex given in kcal mol^-1^ with zero-point-energy corrections.

**Figure S18** Energy decomposition comparison between TS for Hγax and Hδeq abstraction. The largest differences indicating a larger energetic cost in TS Hγax correspond to removal of interactions for residues S274, V322, and the substrate (PCH 438).

**Table S1** NAD(P)H consumption rate (μM min^-1^ per μM CYP108D18) of CYP108D18 with different redox partners in the presence of substrate (1 mM). Background rate of NAD(P)H consumption due to redox partners were measured in the absence of P450 and subtracted from calculated rates (n=2). ^a^NADH was used; ND indicates not detected. The formation of hydroxylated products was confirmed by GC-MS analysis. “Y” indicates detectable product formation, while “N” denotes no product detected.

| **Redox partner combination** | **Substrate** | **NAD(P)H consumption rate ± stdev** | **Product formation** |
| --- | --- | --- | --- |
| CYP108D18 : Spinach Fdx : Spinach FdR (1:10:2) | phenylcyclohexane | 18 ± 1 | Y |
| CYP108D18 : Spinach Fdx : Spinach FdR (1:10:2) | *p*-cymene | ND | N |
| CYP108D18 : Terpredoxin : ArR (1:10:2) | Phenylcyclohexane | 6 ± 1 | N |
| CYP108D18 : Terpredoxin : ArR (1:10:2) | *p*-cymene | 7 ± 2 | N |
| CYP108D18 : Terpredoxin : PdR^a^ (1:10:2) | Phenylcyclohexane | 8 ± 2 | N |
| CYP108D18 : Terpredoxin : PdR^a^ (1:10:2) | *p*-cymene | 10 ± 3 | N |
| CYP108D18 : Pdx : PdR^a^ (1:8:2) | Phenylcyclohexane/*p*-cymene | ND | N |
| CYP108D18 : Cindoxin : FdR (1:8:2) | Phenylcyclohexane/*p*-cymene | ND | N |
| CYP108D18 : Cymredoxin : PdR^a^ (1:8:2) | Phenylcyclohexane/*p*-cymene | ND | N |

**Table S2** Comparison of heme propionate interactions with key residues in CYP108D18, CYP108D1, and CYP108A1.

| **CYP108D18** | **CYP108D1** | **CYP108A1** |
| --- | --- | --- |
| Leu107 | Leu104 | Leu102 |
| His115 | His112 | His110 |
| Arg119 | Arg116 | Arg114 |
| Arg327 | Arg321 | Arg319 |
| Tyr350 | Tyr344 | Tyr342 |
| Ala380 | Ala374 | Trp372 |
| His383 | His377 | His375 |

**Table S3** Comparison of the access channel residues of CYP108D18, CYP108D1 and CYP108A1.

| **CYP108D18** | **CYP108D1** | **CYP108A1** |
| --- | --- | --- |
| Val79 | Val80 | Ile78 |
| Thr81 | Thr82 | Tyr80 |
| Gly85 | Gly86 | Asn84 |
| Ala89 | Ala90 | Met88 |
| Leu103 | Leu100 | Val98 |
| Phe196 | Phe190 | Phe188 |

**Table S4** Comparison of the active site residues of CYP108D18, CYP108D1 and CYP108A1.

| **CYP108D18** | **CYP108D1** | **CYP108A1** |
| --- | --- | --- |
| Val78 | Thr79 | Glu77 |
| Val104 | Val101 | Ile99 |
| Ser106 | Ser103 | Ser101 |
| Val108 | Val105 | Thr103 |
| Ala109 | Gln106 | Ser104 |
| Ile271 | Ile265 | Ala263 |
| Ser274 | Ser268 | Thr266 |
| Ala275 | Ala269 | Ala267 |
| Asp278 | Asp272 | Asp270 |
| Thr279 | Thr273 | Thr271 |
| Val322 | Val316 | Val314 |
| Phe325 | Phe319 | Phe317 |
| Phe422 | Phe416 | Phe414 |
| Val423 | Val417 | Val415 |

**Table S5** AutoDock Vina docking scores of phenylcyclohexane and selected plant-derived hydrophobic compounds within the active site of CYP108D18.

| **Compounds** | **Docking Scores (kcal/mol)** | **Heme spin-state shifts (%)** |
| --- | --- | --- |
| phenylcyclohexane | -8.0 | 96 ± 2 |
| *p*-cymene | -6.9 | 57 ± 6 |
| (*R*)-limonene | -6.9 | 61 ± 2 |
| α-terpineol | -6.2 | 40 ± 2 |
| α-humulene | -8.5 | 10 ± 6 |
| valencene | -8.3 | 44 ± 9 |
| phytol | -8.1 | 21 ± 5 |
| isophyllocladene | -8.8 | < 5 |
| 𝛽-sitosterol | -11.1 | < 5 |
| moronic acid | -12.4 | 25 ± 4 |

# References

1. Bell, S.G., Yang, W., Yorke, J.A., Zhou, W., Wang, H., Harmer, J., et al. (2012) Structure and function of CYP108D1 from *Novosphingobium aromaticivorans* DSM12444: an aromatic hydrocarbon-binding P450 enzyme. *Acta Crystallogr D Biol Crystallogr* **68**: 277–291.
2. Bernhardt, P.V., Chen, K.-I., and Sharpe, P.C. (2006) Transition metal complexes as mediator-titrants in protein redox potentiometry. *J Biol Inorg Chem* **11**: 930–936.
3. Coleman, T., Kirk, A.M., Lee, J.H.Z., Doherty, D.Z., Bruning, J.B., Krenske, E.H., et al. (2022) Different geometric requirements for cytochrome P450-catalyzed aliphatic versus aromatic hydroxylation results in chemoselective oxidation. *ACS Catal* **12**: 1258–1267.
4. Loew, G.H. and Harris, D.L. (2000) Role of the heme active site and protein environment in structure, spectra, and function of the cytochrome P450s. *Chem Rev* **100**: 407–420.
5. Miyamoto, S. and Kollman, P.A. (1992) Settle: An analytical version of the SHAKE and RATTLE algorithm for rigid water models. *J Comput Chem* **13**: 952–962.
6. Roe, D.R. and Cheatham, T.E. (2013) PTRAJ and CPPTRAJ: software for processing and analysis of molecular dynamics trajectory data. *J Chem Theory Comput* **9**: 3084–3095.
7. Ryckaert, J.-P., Ciccotti, G., and Berendsen, H.J.C. (1977) Numerical integration of the cartesian equations of motion of a system with constraints: molecular dynamics of *n*-alkanes. *Journal of Computational Physics* **23**: 327–341.
8. Shahrokh, K., Orendt, A., Yost, G.S., and Cheatham, T.E. (2012) Quantum mechanically derived AMBER‐compatible heme parameters for various states of the cytochrome P450 catalytic cycle. *J Comput Chem* **33**: 119–133.
